# DNA barcodes reveal population-dependent cryptic diversity and various cases of sympatry of Korean leptonetid spiders (Araneae: Leptonetidae)

**DOI:** 10.1038/s41598-022-18666-y

**Published:** 2022-09-15

**Authors:** Jong-Hwa Oh, Sora Kim, Seunghwan Lee

**Affiliations:** 1 Laboratory of Insect Biosystematics, Department of Agricultural Biotechnology, Seoul National University, Seoul, Republic of Korea; 2 Research Institute of Agriculture and Life Sciences, Seoul National University, Seoul, Republic of Korea; 3 Laboratory of Insect Phylogenetics and Evolution, Department of Plant Protection & Quarantine, Jeonbuk National University, Jeonju, Republic of Korea; 4 Department of Agricultural Convergence Technology, Jeonbuk National University, Jeonju, Republic of Korea

**Keywords:** Evolution, Zoology

## Abstract

Leptonetidae are tiny, rarely encountered spiders that mainly inhabit moist environments, such as caves, leaf litter, and rock piles. Because they are microhabitat specialists, most leptonetid species have short-range endemism, and rarely occur in sympatry. Their small size, relatively simple habitus features and reproductive organ structure increase the difficulty of identification. The identification of leptonetids and other spiders may also be time-consuming due to their sexual dimorphism, polymorphism, and lack of diagnostic characteristics in juveniles. DNA barcoding has been used as an effective tool for species identification to overcome these obstacles. Herein, we conducted a test of DNA barcoding based on 424 specimens of Korean Leptonetidae representing 76 morphospecies. A threshold of 4.2% based on maximum intraspecific genetic divergence was estimated to efficiently differentiate the morphospecies. The species assignments tested by five species delimitation methods (ABGD, ASAP, GMYC, PTP, and bPTP) were consistent with the morphological identifications for only 47 morphospecies (61.8%), indicating many cases of cryptic diversity among the remaining morphospecies. Furthermore, sympatry in leptonetids, which are known to be rare, was revealed to be common in South Korea, especially in epigean species. Our results showed that sympatries within families, congeners, and intraclades potentially occur throughout the entire region of Korea.

## Introduction

Spiders of the family Leptonetidae Simon, 1890 currently include 21 genera and 368 species that mainly inhabit the Holarctic region. To date, 57 species in seven genera have been recognized in the Nearctic region, 74 species in eight genera have been identified in the Mediterranean region, and 237 species in eight genera have been identified in Asia^1^.

Leptonetids are small (1–3 mm) spiders that build sheet webs, on which they hang below. They are known to prefer dark and moist microhabitats, such as leaf litter, layered rock piles, mines, and caves^2–7^. This family is well-known to have a strong cave association because more than 50% of the species have only been identified in cave habitats. Many of these cave species have highly troglomorphic morphologies, such as eye reduction, depigmentation, and elongation of the appendages^8^. In contrast, in South Korea, only 17% (9 species out of 52 species) of the species have only been identified in caves, and the remainder have been identified in epigean habitats^2,3,7,9–23^. Subterranean habitats and islands are known as one of the best barriers to geographic isolation, resulting in high endemism in arthropod taxa, including spiders^24–30^. However, within leptonetids, especially within epigean species, the speciation barrier is ambiguous and poorly understood. Because they are rarely seen, the general biology and life history of leptonetid spiders are poorly understood, and only some of the reproductive biology has been reported thus far^4,30,31^. Due to their habitat preferences, most species have a limited distribution range and rarely occur in sympatry, with this characteristic only observed in a few epigean populations^4,5^.

In studies of North American leptonetids, Gertsch suggested intraspecific polymorphism in troglomorphic characters, such as the reduction of eyes and depigmentation in *Neoleptoneta capilla*^32^. Additionally, *Tayshaneta myopica* and *Tayshaneta paraconcinna* presented more complex polymorphisms and genetic diversity that was dependent on the population and habitat type^6,33^. Similar patterns were also found in South Korea, but only between epigean populations. Compared with research performed in North America, studies performed on Korean leptonetids have focused on α-level diversity and treated cases of polymorphism as a separate species^2,3,7,22,23^.

Sexual dimorphism, polymorphisms, and limited morphological information on juveniles or larvae have been obstacles for morphological identification^34,35^. To overcome these obstacles, DNA barcoding has been developed, and it represents an efficient and popular tool for taxonomic investigations for the accurate species identification, cryptic species discovery and biodiversity estimates of animal taxa^36–44^, including spiders^45–62^. DNA barcoding of leptonetids has also been performed based on genetic distance and the Automatic Barcode Gap Discovery (ABGD) method in *Leptonetela* taxa^62^. Their study included numerous species from over a hundred cave populations throughout China and Europe. However, their sampling was mainly based on populations from caves and did not include populations from epigean habitats.

In this study, we (i) tested the utility of DNA barcodes for species identification, delimitation, and new species discovery, (ii) compared morphology-based species delimitation and barcode-based approaches, (iii) searched for types of barriers that lead to genetic diversity and speciation, including epigean leptonetids, by determining the range of species distribution, (iv) provided morphological characters that support results of delimitation methods, (v) detected cases of cryptic species, and (vi) generated a DNA barcode reference library of described species and putative new leptonetid species.

## Material and methods

### Taxon sampling and morphological identification

**Figure 1:**
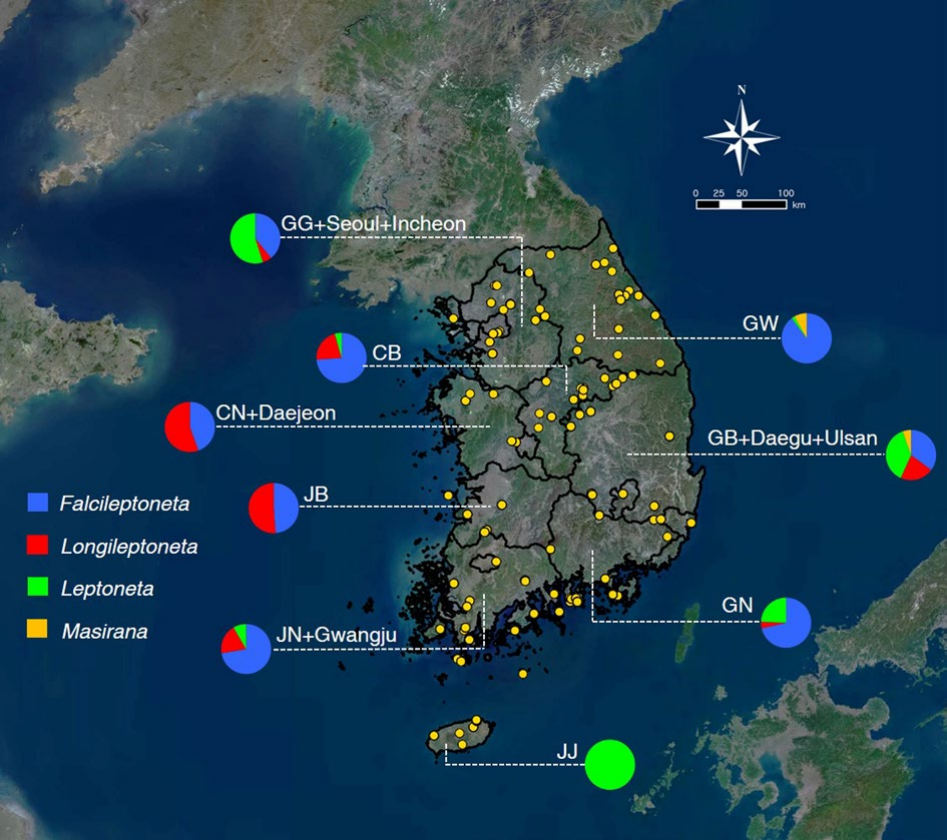
Map with sampling sites for collected specimens used in the present study. Pie charts indicate the proportion of each genus sampled from respective administrative localities. The abbreviations of each administrative district follow: GG, Gyeonggi-do; GW, Gangwon-do; CB, Chungcheongbuk-do; CN, Chungcheongnam-do; JB, Jeollabuk-do; JN, Jeollanam-do; GB: Gyeongsangbuk-do; GN, Gyeongsangnam-do; JJ, Jeju-do. The map was prepared using QGIS 3.22.1 (https://www.qgis.org/ko/site/).

As part of an ongoing revision of the Korean Leptonetidae, we examined approximately 900 specimens (including more than 600 adult specimens) collected from approximately 150 sites in 15 (out of 17) administrative districts, including 22 caves/mines and 11 islands of South Korea (Fig. 1, Supplementary Table S1 online). Samples were mainly collected by net sifting, pit-fall traps, and exploration in caves or mines. Between 1 and 26 individuals were sampled per site, placed directly into 98% ethanol, and stored at − 20 °C. Before DNA extraction, the habitus and the male palp of the specimens were examined under Leica Z16 APO stereomicroscope. Female genitalia were separated from opisthosoma using a microsurgery stiletto knife. Separated genitalia were cleared by heating in 5 ml tubes of lactic acid or 10% KOH solution for 1 h to dissolve extraneous tissue, and then examined and photographed with Olympus BX53 compound microscope. All specimens with vouchers are deposited in the College of Agriculture and Life Sciences, Seoul National University (CALS, SNU, Seoul), National Institute of Biological Resources (NIBR, Incheon), and Yangpyeong Insect Museum (YIM, Yangpyeong), Republic of Korea. The sampling map was generated with QGIS 3.22.1 (https://www.qgis.org/ko/site/). Digital editing of the figures, microscopic photographs, and maps was prepared using Adobe Photoshop CC 2018 (Adobe Systems Incorporated, https://www.adobe.com/products/photoshop.html).

### DNA extraction, PCR amplification, and Sequencing

Genomic DNA was extracted from muscle tissue by grinding usually 2–4 legs, or whole body except the abdomen using DNeasy Blood and Tissue kit (QIAGEN, Hilden, Germany) following the manufacturer’s protocols. For PCR amplification for mitochondrial cytochrome oxidase subunit I (mtCOI, ~ 901 bp), we used universal primers or primers developed and used in arachnid taxa. Primer combinations we used in this study were forward LCO1490 (5’-GGTCAACAAATCATCATAAAGATATTGG-3’)^63^ with reverse HCO2568 (5’-GCTACAACATAATAAGTATCATG-3’)^64^ or HCOoutout (5’-GTAAATATATGRTGDGCTC-3’)^65^. Amplification was performed using AccuPower PCR Premix (Bioneer, Daejeon, Republic of Korea) following the standard protocols. The PCR condition consisted to initial denaturation at 95 °C for 2 min, followed by 35 cycles of denaturation at 95 °C for 30 s, annealing at 45–50 °C for 30 s, extension at 72 °C for 45 s, and a final extension at 72 °C for 10 min. Successfully amplified PCR products were checked in 1.2% agarose gels and were purified and sequenced at BIONICS, Inc. (Seongdong-gu, Seoul, Republic of Korea).

### Sequence analysis, genetic distance, phylogenetic analysis

Raw sequences of the COI region were assembled and edited using SeqMan^TM^II (version 5.01, 2001; DNA-star™). We eliminated poor quality and short DNA sequences in order to prevent any risk of confusion or errors, and ended up with 411 COI sequences. 13 additional sequences were downloaded from NCBI (see Supplementary Table S1 for accession numbers). Therefore, a total of 424 sequences were carried out for alignment using MAFFT version 7^66^ through the EMBL-EBI online portal using the L-INS-i method. The sequences were deposited in GenBank (ON041801–ON042211, Supplementary Table S1). The sequence data were then combined using SequenceMatrix windows ver. 1.8^67^. To calculate intra/interspecific pairwise genetic distances, we implemented the Kimura-2 parameter (K2P) model using MEGA 7.0^68^. Finally, haplotype data were generated in DnaSP6.12^69^ to identify the distinct haplotypes.

For phylogenetic analysis, Maximum likelihood (ML), and Bayesian Inference (BI) were generated in order to test monophyletic criteria of species delimitation. Aligned sequences were carried out to PartitionFinder v2.1.1^70^ to choose the best fit model for the analysis. RaxML GUI v2.0 was used under GTR + GAMMA model for ML tree analysis^71,72^. IQ-tree was also conducted as ML tree analysis using IQ-TREE version 1.6.7.1^73^. BI tree was constructed using MrBayes v3.2.7^74^. The Markov chain Monte Carlo search for the data matrix ran four chains for 10,000,000 generations with 25% burn-in, and sampling every 100 generations. Finally, the phylogenetic tree was visualized in FigTree v1.4.4^75^.

### Barcoding, species delimitation, and MOTUs estimation

To estimate the number of Molecular Operational Taxonomic Units (MOTUs), we used five species delimitation methods: Automatic Barcode Gap Discovery (ABGD)^76^, Assemble Species by Automatic Partitioning (ASAP)^77^, Generalized Mixed Yule-coalescent (GMYC)^78^, Poisson-Tree-Processes (PTP), and Bayesian implementation of the PTP (bPTP)^79^.

ABGD method delimit hypothetical species by calculating the pairwise distance based on the barcode gap. The ABGD analysis was performed online (https://bioinfo.mnhn.fr/abi/public/abgd/) under Jukes-Cantor (JC69), Kimura 2-parameter (K2P), and uncorrected distance (p-distance) model with relative gap width (X = 1.5).

ASAP is a recently developed method designed to propose species partitions using a hierarchical clustering algorithm based on pairwise genetic distances. The aligned sequences were submitted online (https://bioinfo.mnhn.fr/abi/public/asap/) under models the same as the ABGD method in default settings.

In the GMYC analysis, we used BEAST v2.6.6^80^ to obtain an ultrametric tree, under a strict molecular clock model. In the prior, we used the Yule speciation model running 20 million generations, sampling every 1000 generations. We further checked for stationarity and determined burn-in using TRACER v1.7.2^81^, and then discarded as 15% burn-in, 0.5 posterior probability using TreeAnnotator^80^. For the GMYC analysis, the ultrametric tree was then carried out in RStudio (https://www.r-project.org/) using the “splits” package^82^.

PTP is a coalescent-based delimitation method that requires a phylogenetic input tree, and bPTP is an updated version of the original PTP, adding Bayesian support (BS) values to delimited species on the input tree. We used the BI tree as the input tree for PTP and bPTP analyses, implemented online (https://species.h-its.org/ptp), running 100,000 MCMC generations, with a thinning of 100, burn-in of 0.1, and removing the outgroups for improved results.

## Results

### Morphological identification

Based on the morphological examination, 75 morphospecies in four genera (*Falcileptoneta*, *Leptoneta*, *Longileptoneta*, and *Masirana*) of leptonetids were identified from 409 specimens (Supplementary Table S2 online). Additionally, two individuals of Telemidae from South Korea, were also included as an outgroup.

Although leptonetids are known as microhabitat specialists and highly localized, many species, especially epigean leptonetids, were sampled from various distant populations, indicating that they are more widely distributed than expected around the Korean Peninsula (*Falcileptoneta odaesanensis, Leptoneta taeguensis, Leptoneta chilbosanensis, Leptoneta spinipalpus, Longileptoneta weolakensis, Longileptoneta songniensis,* and *Longileptoneta* sp3). In some cases, the same species were distributed across the islands (*Falcileptoneta* sp17, *Falcileptoneta* sp21, *Leptoneta namhensis,* and *Leptoneta paikmyeonggulensis*).

### Genetic distance divergence of species identification

A haplotype data analysis revealed 200 distinct haplotypes (Supplementary Data S1 online). The average K2P genetic divergence was 20.74% across all specimens of the dataset. The mean of intraspecific K2P genetic divergence was 1.27% (ranging from 0 to 18.41%), with an increase in divergence between congeners of approximately 13 times to a value of 16.64% (ranging from 14.49 to 21.22%) on average (Table 1). However, the interspecies divergence within genera varied from 10.46% to 21.22%: *Falcileptoneta* (mean = 20.40%), *Longileptoneta* (mean = 10.46%), *Leptoneta* (mean = 21.22%), and *Masirana* (mean = 14.49%). Detailed values of each genus are provided in Table 2. The number of sampled sites, sampled specimens, and intraspecific genetic divergences of each morphospecies are listed in Supplementary Data S2.

**Table 1:** K2P genetic distance depends on the species and genus of Leptonetidae.

Comparisons	Mean of K2P genetic distance (%)
Overall	20.74
Within-species	1.27
Within- genus	16.64

**Table 2:** K2P genetic distances between species within each genus.

Comparisons	Mean (%)	Minimum (%)	Maximum (%)
*Falcileptoneta*	20.40	4.50	31.0
*Longileptoneta*	10.46	3.60	17.10
*Leptoneta*	21.22	9.70	29.20
*Masirana*	14.49	11.10	16.80

A threshold was determined to evaluate the number of MOTUs in Leptonetidae, and the maximum value of intraspecific K2P genetic divergence was less than 4.2%, with 11 morphospecies presenting a maximum intraspecific divergence over 4.2% (Fig. 3).

### MOTU estimation

**Figure 2:**
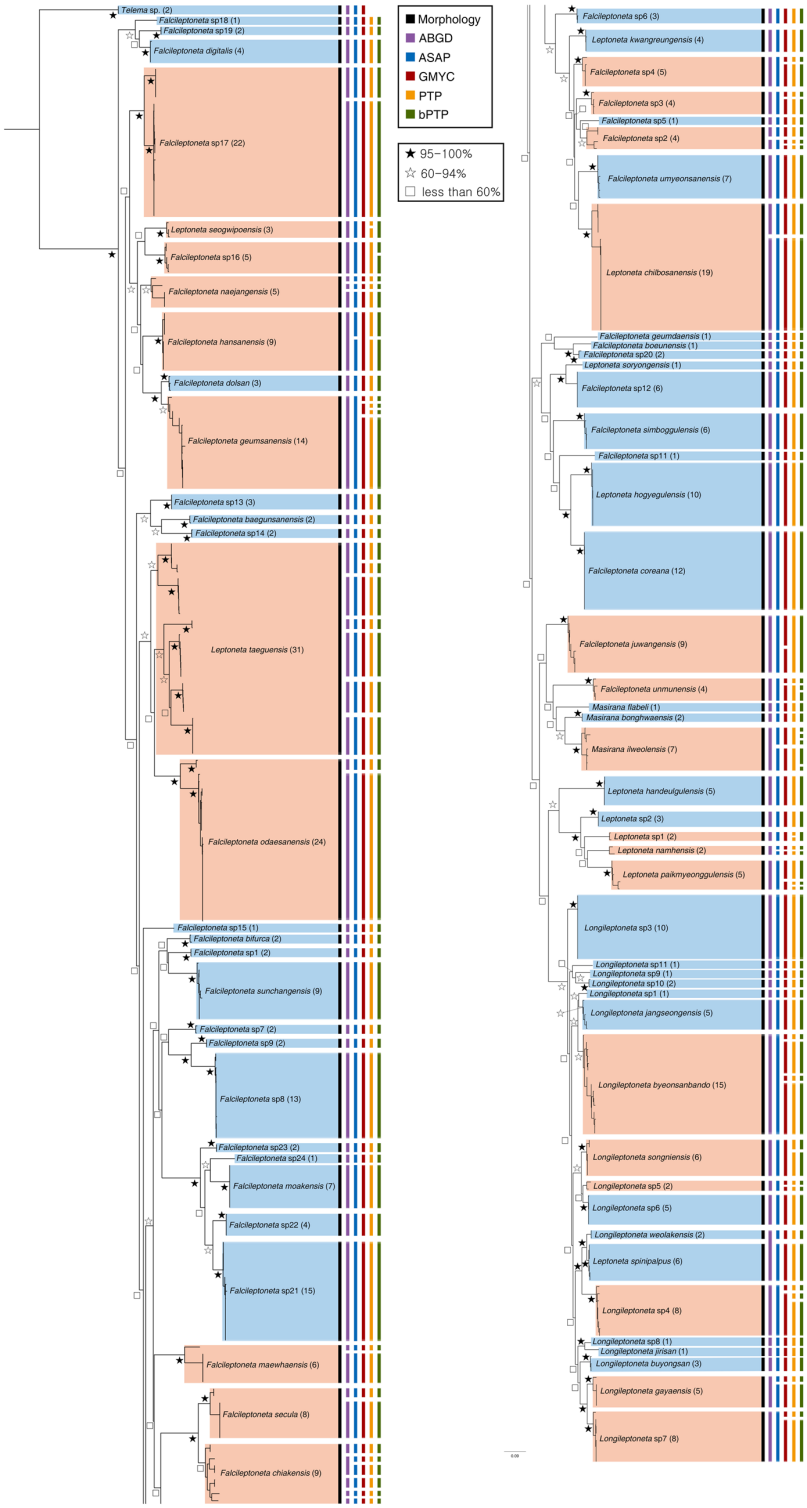
Maximum Likelihood (ML) tree of 424 COI sequences, including morphological analysis and results of five different species delimitation methods. Color bars indicate the results of each delimitation method. In the ML tree, blue panels indicate that all species delimitation methods match with morphological identification, while red panels show one or more methods disagree.

The species delimitation methods of ABGD, ASAP, GMYC, PTP, and bPTP yielded 92, 98, 112, 117, and 120 MOTUs, respectively. Color bars in the ML tree indicate the results of MOTUs delineated by different methods (Fig. 2; IQ-tree in Supplementary Fig. S1). Compared with the morphological identification method, species delimitation methods are mostly focused on the population level rather than morphological appearance (see Supplementary Table S3 online). Phylogenetic tree-based methods generally exhibit greater oversplitting compared with genetic distance-based methods. As a result, 47 MOTUs, which accounted for 61.8% of the total, matched the morphological delimitation of the species.

An ABGD analysis of each JC69, K2P, and p-distance substitution model produced nonidentical MOTUs, resulting in 82, 92, and 91 MOTUs that with 85.5%, 85.5%, and 82.9% consistency with the morphology, respectively (all *P* = 0.035938 (for more details, see Supplementary Table S4 online)). Considering the morphological identifications and comparisons with other species delimitations used in this study, we display the ABGD results based on the K2P substitution model. Six morphospecies, *Falcileptoneta secula*, *Falcileptoneta maewhaensis*, *Falcileptoneta* sp2, *Falcileptoneta odaesanensis*, *Falcileptoneta* sp17, and *Leptoneta chilbosanensis* were split into two MOTUs, *Falcileptoneta chiakensis* was split into five MOTUs, and *Leptoneta taeguensis* was split into six MOTUs by ABGD. On the other hand, *Longileptoneta weolakensis* and *Leptoneta spinipalpus* were merged into a single MOTU.

Compared with the ABGD method, the ASAP analysis produced identical MOTU results on each JC69, K2P, and p-distance substitution model, which all yielded 98 MOTUs with ASAP scores of 7.00, 9.50, and 6.50, respectively (for more details, see Supplementary Table S5 online). Compared with ABGD, additional MOTUs were observed in seven morphospecies: *Falcileptoneta chiakensis*, *Falcileptoneta hansanensis*, *Longileptoneta gayaensis*, *Longileptoneta weolakensis*, *Leptoneta spinipalpus, Leptoneta namhensis*, and *Masirana ilweolensis*.

The GMYC analysis produced more MOTUs, and it likely oversplit them into 112 MOTUs. Compared with the ABGD and ASAP methods, 12 species were split into additional MOTUs by the GMYC method: *Falcileptoneta* sp2, *Falcileptoneta* sp3, *Falcileptoneta* sp4, *Falcileptoneta juwangensis*, *Falcileptoneta geumsanensis*, *Falcileptoneta unmunensis*, *Longileptoneta byeonsanbando*, *Longileptoneta* sp3, *Longileptoneta* sp4, *Longileptoneta* sp6, *Leptoneta taeguensis*, and *Leptoneta paikmyeonggulensis*. Conversely, *Falcileptoneta hansanensis* was recovered as a single MOTU in the GMYC analysis.

The PTP analysis yielded 117 MOTUs, which was similar to the GMYC delineation. However, the MOTUs of the five morphospecies were split by the PTP method from GMYC: *Falcileptoneta geumsanensis*, *Falcileptoneta hansanensis*, *Longileptoneta* sp4, *Leptoneta* sp1, and *Leptoneta paikmyeonggulensis*. Additionally, *Falcileptoneta juwangensis* was recovered as a single MOTU from GMYC.

The bPTP produced the most MOTUs among the methods we used in this study, and it yielded 120 MOTUs. Although bPTP recovered *Leptoneta* sp1 into a single MOTU, which was split by the PTP method, it split the other morphospecies, e.g., *Falcileptoneta unmunensis* was split into three MOTUs, *Leptoneta paikmyeonggulensis* was split into four MOTUs, and *Masirana ilweolensis* was split into five MOTUs.

### Population-dependent cryptic diversity

**Figure 3:**
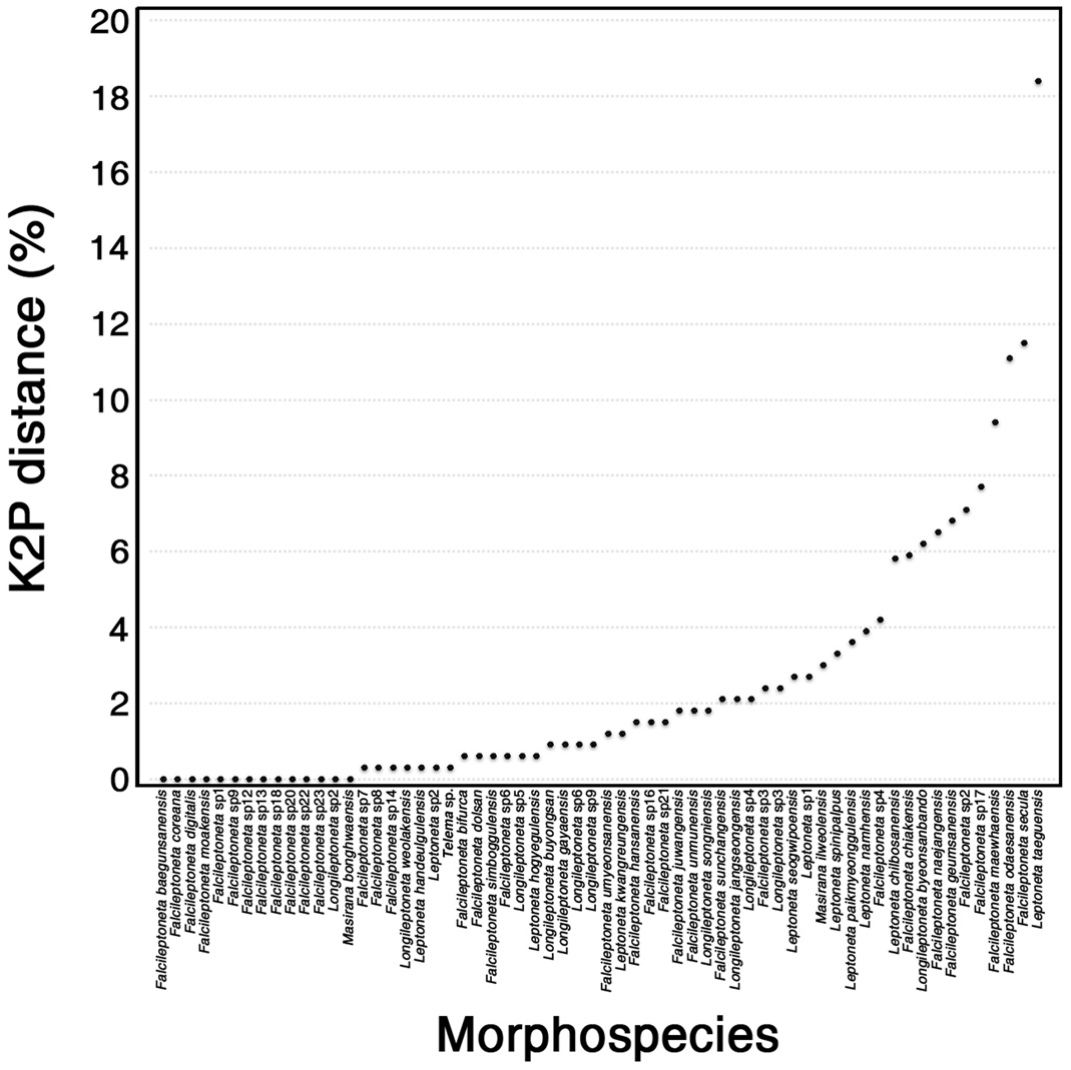
Maximum intraspecific divergences (%) based on Kimura 2-parameter model from the dataset. Morphospecies having a single specimen are not included.

The threshold based on the maximum intraspecific K2P genetic divergence was estimated at 4.2% from the dataset, with 11 morphospecies showing divergence over the maximum estimated threshold (5.8–18.4%) (Fig. 3). In particular, nine morphospecies were split into multiple MOTUs by all five species delimitation methods. The morphospecies had a maximum divergence of over 4.2% and their delimitation results were mainly population dependent, especially the epigean leptonetids.

**Figure 4:**
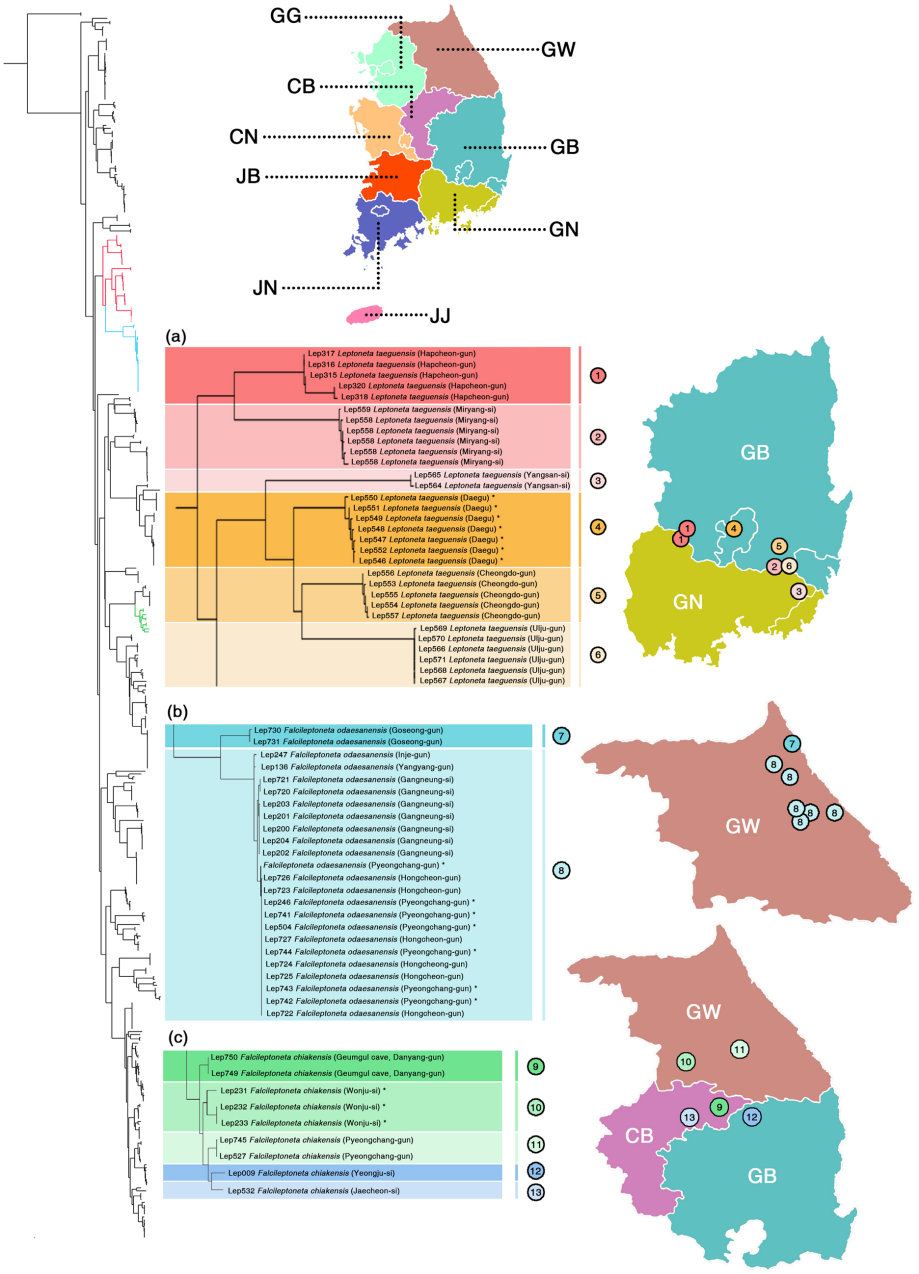
Cryptic species between various populations of epigean leptonetids with a map of sampled localities. All maps were prepared using QGIS 3.22.1 (https://www.qgis.org/ko/site/). (**a**) *Leptoneta taeguensis* from (1) Hapcheon-gun, (2) Miryang-si, (3) Yangsan-si, (4) Daegu, (5) Cheongdo-gun, and (6) Ulju-gun; (**b)** *Falcileptoneta odaesanensis* from (7) Goseong-gun, (8) Inje-gun, Yangyang-gun, Gangneung-si, Pyeongchang gun, and Hongcheon-gun, (**c**) *Falcileptoneta chiakensis* from (9) Danyang-gun, (10) Wonju-si, (11) Pyeongchang-gun, (12) Yeongju-si, and (13) Jaecheon-si. Asterisks represent the individuals collected from type localities.

**Figure 5:**
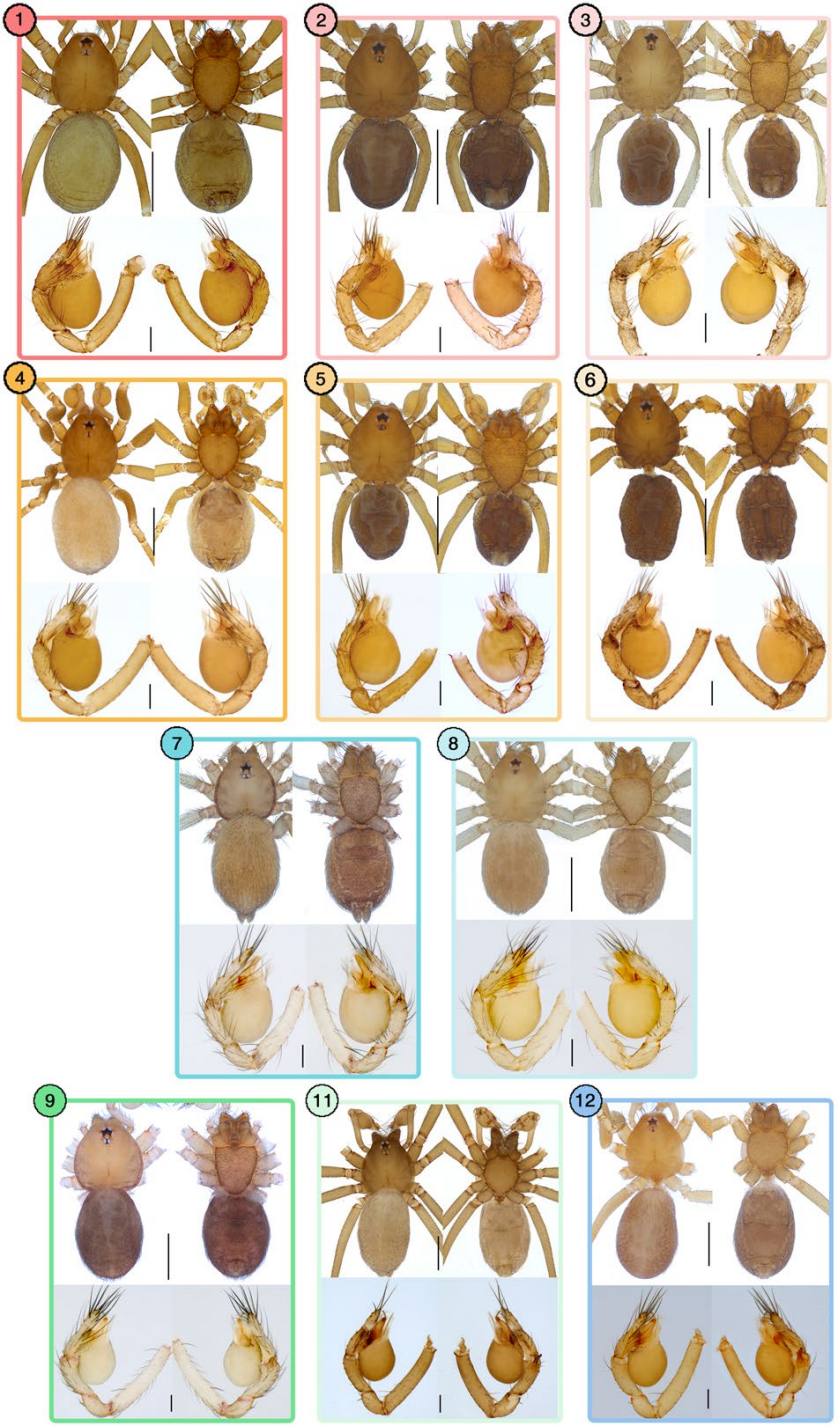
Morphological photographs of each cryptic species from Fig. 4. upper panels: habitus (dorsal and ventral view); lower panels: left palp (prolateral and retrolateral view), (6) for right palp (retrolateral and prolateral view). The numbers of each panel match the numbers from Fig. 4. Scale bars: upper panels = 0.5 mm, lower panels = 0.1 mm.

First, among the epigean leptonetids (Fig. 4), the morphospecies *Leptoneta taeguensis* was split into six to seven MOTUs (Fig. 4a), *Falcileptoneta odaesanensis* was into two MOTUs (Fig. 4b), and *Falcileptoneta chiakensis* was split into five to six MOTUs (Fig. 4c) depending on each populational locality by the species delimitation methods used in this study (morphological details on Fig. 5, see the original description of the morphospecies *Falcileptoneta chiakensis*^18^).

**Figure 6:**
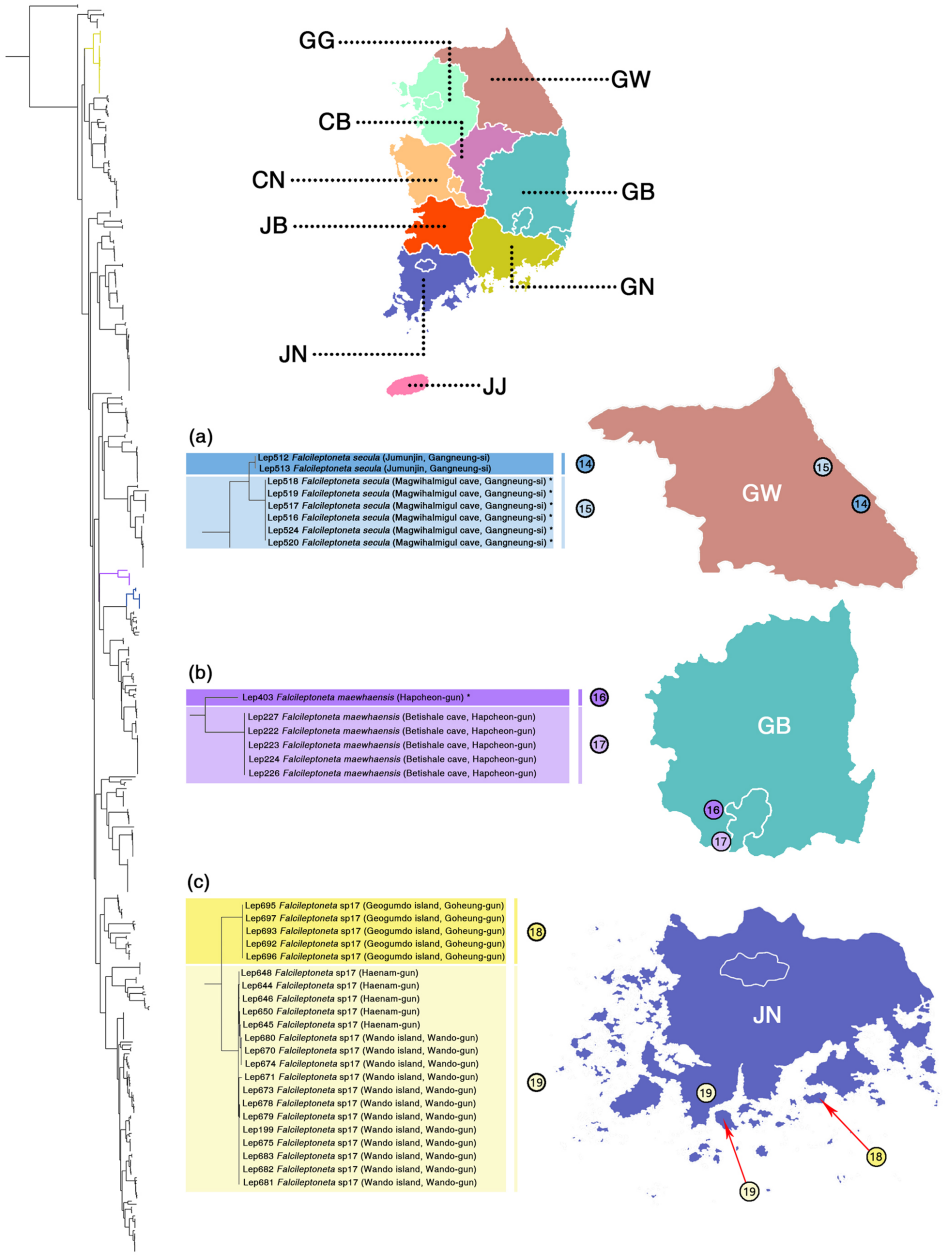
Cryptic species between cave populations vs. epigean populations, and mainland populations versus island populations with a map of sampled localities. All maps were prepared using QGIS 3.22.1 (https://www.qgis.org/ko/site/). (**a**) *Falcileptoneta secula* from (14) Jumunjin (Gangneung-si) and (15) Magwihalmigul cave (Gangneung-si), (**b**) *Falcileptoneta maewhaensis* from (16) Gaya-myeon (Hapcheon-gun), and (17) Betishale cave (Hapcheon-gun), (**c**) *Falcileptoneta* sp17 from (18) Geogumdo island (Goheung-gun), and (19) Mt. Duryunsan (Haenam-gun), Wando island (Wando-gun). Asterisks represent the individuals collected from type localities.

**Figure 7:**
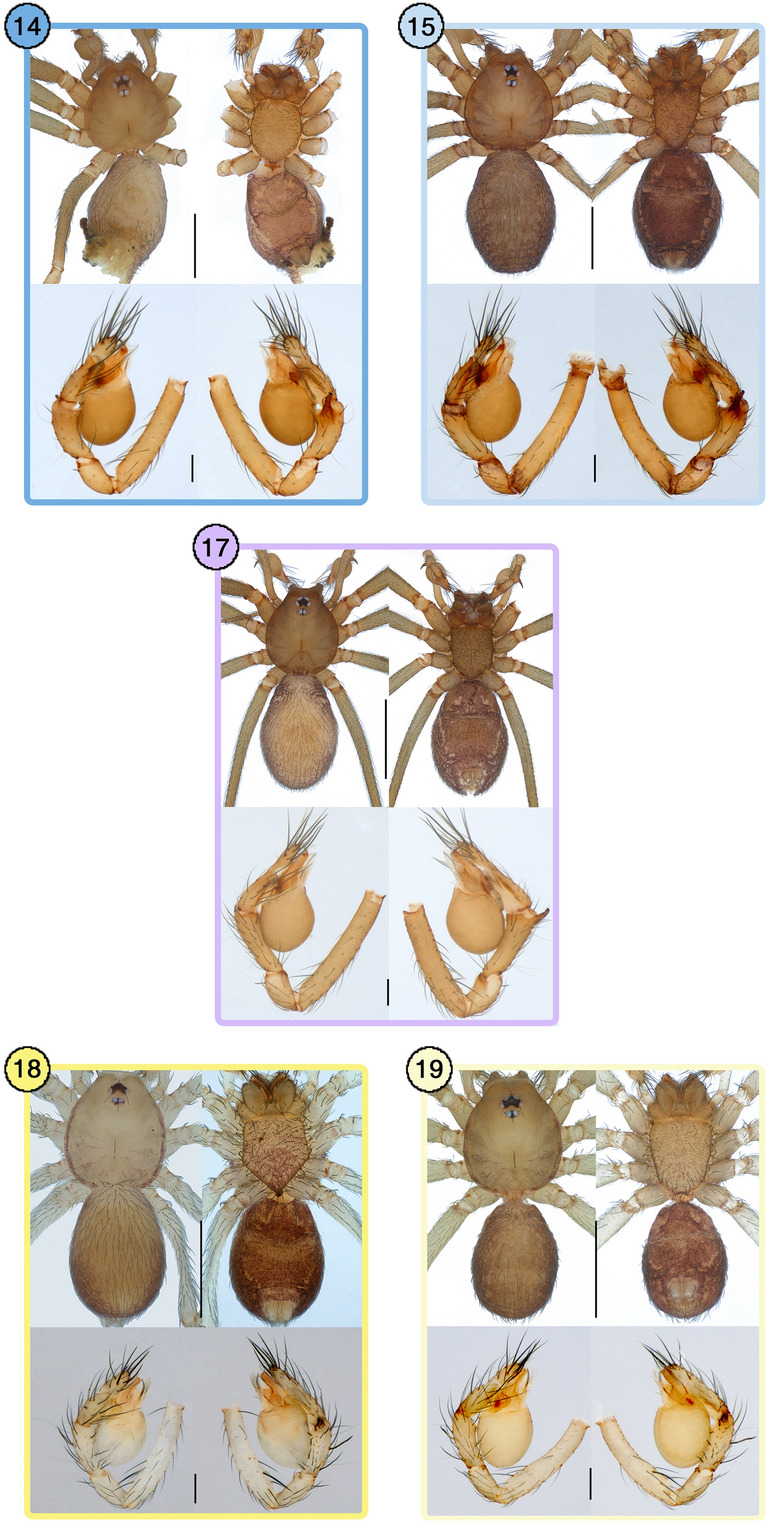
Morphological photographs of each cryptic species from Fig. 6. upper panels: habitus (dorsal and ventral view); lower panels: left palp (prolateral and retrolateral view). The numbers of each panel match the numbers from Fig. 6. Scale bars: upper panels = 0.5 mm, lower panels = 0.1 mm.

Second, in troglophilic leptonetids (Fig. 6), separated MOTUs were detected between the cave population and epigean population, although those populations were not readily diagnosable (*Falcileptoneta secula* and *Falcileptoneta maewhaensis*) (Fig. 6a,b, morphological details on Fig. 7, see the original description of *Falcileptoneta maewhaensis*^21^). As an exception, the cave population and epigean population of the morphospecies *Falcileptoneta simboggulensis* resulted in merged MOTU.

Finally, species that were geographically isolated by islands showed both single and double MOTUs by all five species delimitation methods (Fig. 6c, morphological details in Fig. 7). The population from the mainland of South Korea (Haenam-gun, Jeollanam-do), resulted in a MOTU merged with the population from the island nearby (Wando Island, Wando-gun, Jeollanam-do), while the MOTU that split from the population were distributed relatively distant from the island (Geogumdo Island, Goheung-gun, Jeollanam-do).

### Widely distributed species, and congeneric sympatry in leptonetids

**Figure 8:**
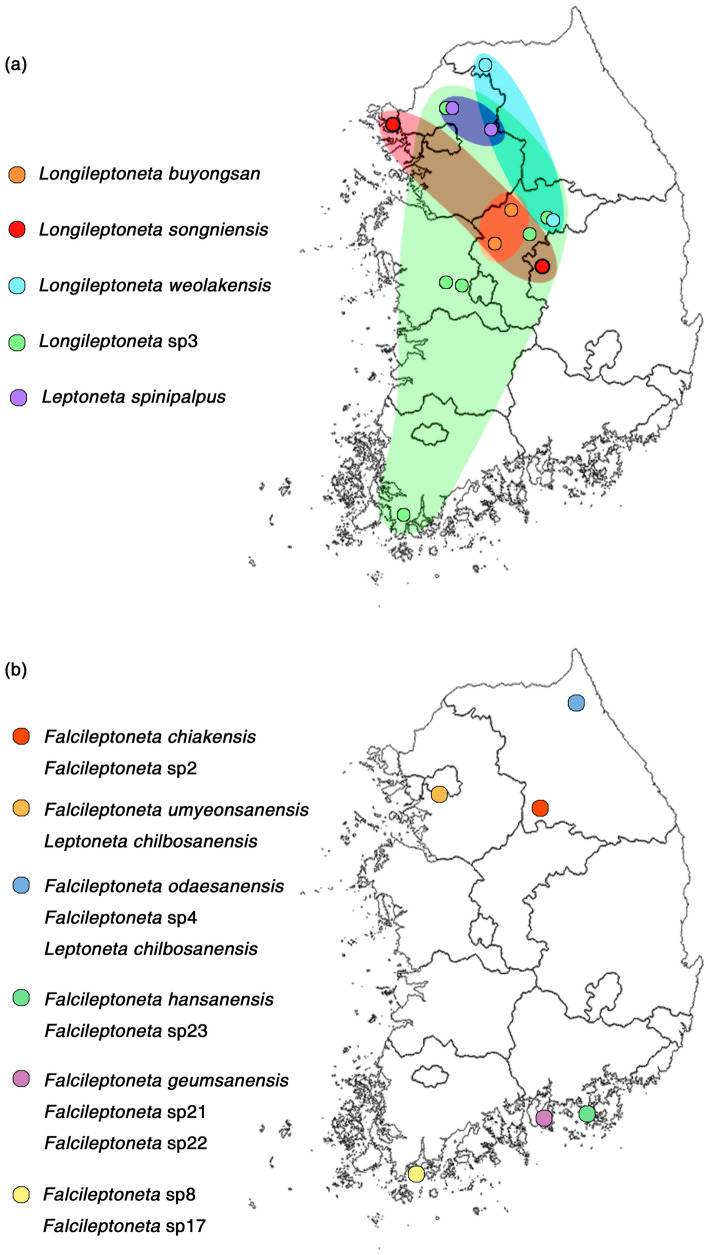
Maps showing sympatric localities between congeneric species. The maps were prepared using QGIS 3.22.1 (https://www.qgis.org/ko/site/). Members of *Longileptoneta* are mainly wider distributed compared to species of *Falcileptoneta*, suggesting a possibility that this genus may have potentially more sympatric distributions.

First, in many cases, particularly for members of the genus *Longileptoneta*, a single MOTU was sampled between distant populations, indicating that many of the species are distributed in a large range and may present sympatry within congeners (Fig. 8a): (i) *Longileptoneta* sp3, which is a potentially new species distributed nationwide in the western part of South Korea (Pocheon-si, Jaecheon-si, Daejeon, Haenam-gun); (ii) *Longileptoneta songniensis*, which was previously only found at Mt. Songnisan (Boeun-gun, Chungcheongbuk-do) but has also been found on Ganghwado Island (Incheon); (iii) *Longileptoneta weolakensis*, which was previously only found at Mt. Weolaksan (Jecheon-si) but has also been found on Mt. Baegunsan (Pocheon-si, Gyeonggi-do).

Second, some cases revealed that different populations across the islands were merged into a single MOTU: (i) *Falcileptoneta* sp17 from Mt. Duryunsan (Haenam-gun, Jeollanam-do) and Wando Island (Wando-gun, Jeollanam-do) was different from that on Geogumdo Island (Goheung-gun, Jeollanam-do) (Fig. 6c); (ii) *Falcileptoneta* sp21 from Namsan Park (Goseong-gun, Gyeongsangnam-do) and Namhae Island (Namhae-gun, Gyeongsangnam-do); (iii) *Longileptoneta songniensis* from Mt. Songnisan (Boeun-gun, Chungcheongbuk-do) and Ganghwado Island (Incheon); and (iv) *Leptoneta paikmyeonggulensis* from Namhae Island (Namhae-gun, Gyeongsangnam-do) and Dolsan Island (Yeosu-si, Jeollanam-do) resulted in single MOTUs.

Finally, two or more species of epigean populations belonging to the genus *Falcileptoneta* found at the same locality were detected (Fig. 8b): (i) *F. chiakensis* and *Falcileptoneta* sp2 from Mt. Chiaksan (Wonju-si, Gangwon-do); (ii) *Falcileptoneta umyeonsanensis* and *Leptoneta chilbosanensis* on Mt. Kwanaksan (Seoul); (iii) *Falcileptoneta odaesanensis*, *Falcileptoneta* sp4 and *Leptoneta chilbosanensis* on Mt. Seoraksan (Inje-gun, Gangwon-do); (iv) *Falcileptoneta hansanensis* and *Falcileptoneta* sp23 from Mt. Mireuksan (Tongyeong-si, Gyeongsangnam-do); (v) *Falcileptoneta geumsanensis*, *Falcileptoneta* sp21 and *Falcileptoneta* sp22 in Namhae Island (Namhae-gun, Gyeongsangnam-do); and (vi) *Falcileptoneta* sp8 and *Falcileptoneta* sp17 from Wando Island (Wando-gun, Jeollanam-do). However, in cave habitats, no sympatry was found.

### Sympatry in intraclade species

**Figure 9:**
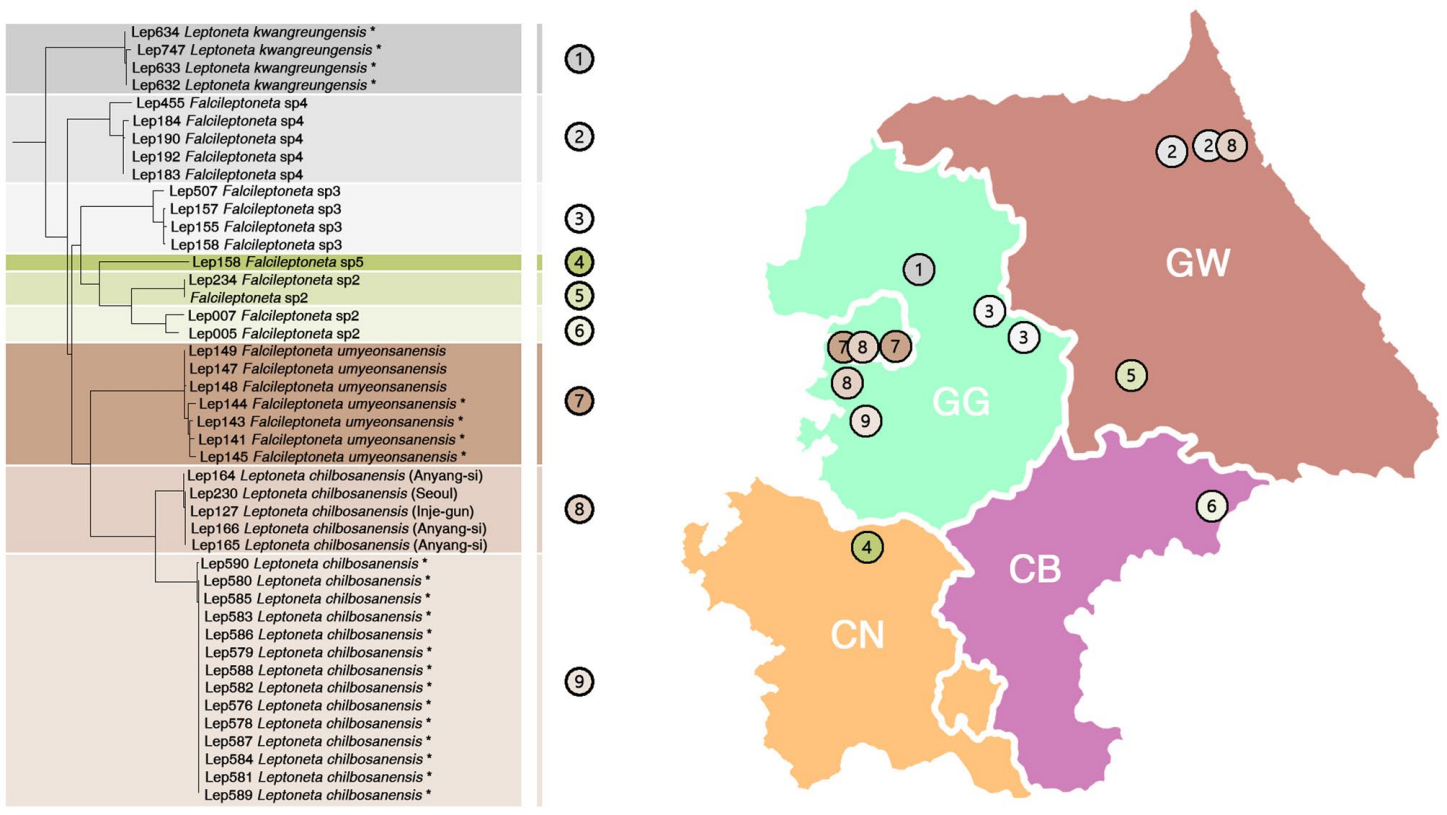
Intraclade sympatry occurring in central region of Korean peninsula. The map was prepared using QGIS 3.22.1 (https://www.qgis.org/ko/site/).

The clade that includes *Falcileptoneta* sp2, *Falcileptoneta* sp3, *Falcileptoneta umyeonsanensis*, *Falcileptoneta* sp4, *Falcileptoneta* sp5, *Leptoneta kwangreungensis*, and *Leptoneta chilbosanensis* has sharp tooth-like tibial apophyses in common. The identification between those species is quite challenging, although they can be diagnosed by the thickness and length of the tibial apophysis of the male palp, spotted patterns of the abdomen, and length of the body. Most of the species appear to present short-range endemism and have only been found in a small range of habitats (one or two mountain ranges). However, clade 8 (Fig. 9) was detected as a cryptic species of *Leptoneta chilbosanensis*, had a large-range distribution and was collected from three administrative districts. Unlike other morphospecies in which cryptic species were detected depending on the population-level, cryptic species of this group were found to be nonpopulation dependent. Moreover, the population from Mt. Surisan (Anyang-si, Gyeonggi-do), one individual among the population of *Falcileptoneta umyeonsanensis* from Mt. Gwanaksan (Seoul), and one individual among the population of *Falcileptoneta* sp4 from Mt. Seoraksan (Inje-gun, Gangwon-do) were all merged to a single MOTU and shared habitat with numerous intraclade species populations.

## Discussion

Accurate identification of leptonetid species can be challenging due to their small body size, similar habitus among different species, and limited morphological information, such as the structure of the male palp. In particular, morphological identification using female and juvenile specimens is problematic due to the lack of diagnosable morphological characteristics, including female genitalia^4,5,33^. Therefore, we tested the utility of DNA barcodes in species identification based on intraspecific divergences and five species delimitation methods (ABGD, ASAP, GMYC, PTP, and bPTP) using Korean leptonetids. Consequently, our results showed some cases of population-dependent cryptic diversity, both observed in short-range (one or two mountain ranges) and large-range distributions (at least including a range of three administrative districts), and at the same time, various types of sympatry, which are rare in this family.

In some spider lineages, especially in several haplogyne spiders such as Hypochilidae, the intraspecific genetic divergence value is extremely high and can exceed 15%^83,84^. Likewise, our results also revealed an extreme divergence of intraspecific genetic distances that exceed 18% (in *Leptoneta taeguensis*), with variations mainly observed at the population level. However, by using species delimitation methods, morphospecies with high intraspecific divergence were split into multiple MOTUs, resulting in cryptic species detection. These results may suggest that cross-validation between intraspecific divergence and species delimitation methods is needed to delineate species boundaries using DNA barcodes. In our study, Korean leptonetids were delineated as a threshold value of 4.2%, which was estimated based on the maximum intraspecific divergences, could serve as an efficient standard for preliminary species delimitation. In particular, the Korean Peninsula includes unique and diverse geographical structures, such as having thousands of caves^85^, presenting a land area that consists of over 70% mountains, and having over 3500 islands off the coast^86^, which may cause frequent geographic isolations. Additionally, it has relatively strong seasonal fluctuations and rich environmental diversity and biodiversity, with over 56,000 biological species^87^, including approximately 2200 endemic species^88^. Therefore, given the different environments around the world, and differences in intraspecific genetic divergences in other spider taxa, this value can be specifically applied to Korean Leptonetidae while other fauna should be further discussed.

In our study, we used various species delimitation methods that are considered the most reliable. ABGD is a genetic distance-based method that is one of the most popular barcode-gap methods^43–46,49,62^, while GMYC, PTP, and bPTP are tree-based delimitation methods used for spider lineages^45,46,49,61,89^ and other arthropod members^43,44^. In most studies of species delimitation methods, phylogenetic tree-based delimitation methods tend to be more sensitive and present more splits into multiple MOTUs compared to barcode gap-based methods, and similar results were obtained in our study (Fig. 2, Supplementary Table S3 online). Tree-based species delimitation methods, especially coalescent-based methods, such as GMYC or BP&P, have been constantly introduced because they are affected by the population structure in the dataset and oversplit taxa, especially when a single gene is applied^45,46,50,89,90^. Because a single gene was applied here, we concede at least four congruent MOTU results among the five different species delimitation methods used in this study as a hypothetical cryptic species (Supplementary Data S3). These results may be important for increasing our understanding of biodiversity and evolution and connecting taxonomy and phylogenetic studies^91^. In further studies, however, a combination of effective markers, such as ribosomal or nuclear genes, would be sufficient for species-level delimitation, as like other arthropods^45,50,59,92–94^.

Morphospecies that resulted in MOTU splits by species delimitation methods and presented high genetic divergence depending on the population level (*Falcileptoneta secula*, *Falcileptoneta odaesanensis*, *Falcileptoneta maewhaensis*, *Falcileptoneta* sp17, and *Leptoneta taeguensis*) were shown to belong to the genus *Falcileptoneta* (Figs. 4, 5, 6, and 7). Compared to other Korean leptonetid genera, especially *Longileptoneta*, species in *Falcileptoneta* tended to have more dispersal-limited endemism. Our sampling data around South Korea indicate that in most cases, many individuals of *Falcileptoneta* species were sampled but in a small range, while few individuals of *Longileptoneta* species were sampled but in larger ranges than expected (Fig. 8a). Based on the analysis of the sampling data, we identified the phenomenon that species in the genus *Longileptoneta* mainly tend to spread out while *Falcileptoneta* gathers.

Leptonetids are known to be dispersal-limited spiders and prefer specific types of microhabitats, such as leaf litter, caves, and mines, creating distributional patterns as ‘narrow endemism’^5,33^. As demonstrated in many other arachnid lineages, the biological traits of restricted dispersion ability and high microhabitat specialization generally lead to biogeographic histories dominated by vicariance, with few dispersal events^95–97^. Additionally, islands and caves play a role in vicariance, thus causing high endemism in arthropod lineages^98–102^. Throughout this study, we found that a considerable number of leptonetid species have expanded their distributions and are being found beyond restricted zones, such as caves and islands, and they also occur in sympatries between different genera, species, and intraspecific clades (Figs. 8, 9). The details of the biogeographical mechanisms leading to these sympatries and a discussion of the phylogenetic relationships will be outlined in a future study (under preparation).

Single morphospecies between the cave population and epigean population near the cave mainly resulted in a split of MOTUs in the species delimitation methods we used in this study (Fig. 5a, b). Ledford and colleagues discussed similar cases in *Tashaneta* species based on multigene phylogeny, and they treated these cases as intraspecific polymorphisms despite furcation on phylogenetic tree^33^. Similarly, our results also showed that populations between the epigean habitat and cave habitats had a high intraspecific genetic divergence, ranging up to a maximum of 11.5%, with a split of MOTUs. However, an exceptional case in this study showed that, *Falcileptoneta simboggulensis*, which is known as a troglobitic spider that has only been found in Simboggul Cave was merged to a single MOTU with epigean individuals which was sampled near the cave (with 0.2% of maximum intraspecific divergence), indicating that this cave does not function as a barrier of gene flow between the cave and epigean populations. Because we lack morphological data for epigean *F. simboggulensis*, additional sampling, especially for male specimens, is needed in future studies.

In the traditional taxonomy of spiders, structures of the female genitalia and the male palp have been key features for species identification. However, many cases in our results showed that species that are indistinguishable based on the male palp resulted in a split of MOTUs depending on the population level. Rather, the shape of the sternum, patterns in the abdomen, and the ratio between the length of the body and leg were somewhat diagnosable from dependent MOTUs (Figs. 5, 7).

In our study, we included several species in the genus *Leptoneta*. However, the phylogenomic and biogeographic study of the family Leptonetidae shows that *Leptoneta* species are restricted in Mediterranean Europe, and all of the species in *Leptoneta* are morphologically, and geographically misplaced^5^. Although Seo transferred many species of *Leptoneta* to *Falcileptoneta* (2015)^18^, more than ten species of Korean leptonetids remain in this genus. Thus, accurate identification based on morphological and phylogenetic studies of misplaced species, especially *Leptoneta*, would be important to better understanding the systematics, biogeography, and evolution of the family Leptonetidae (under preparation).

## Supplementary Information

Supplementary Information 1.

Supplementary Information 2.

Supplementary Information 3.

## Data Availability

Accession Codes: The COI sequences generated and analyzed during the current study are available in the Genbank repository (https://www.ncbi.nlm.nih.gov/genbank/), from ON041801 to ON042211.
