# Supplementary figures and images for: DNA barcodes reveal population-dependent cryptic diversity and various cases of sympatry of Korean leptonetid spiders (Araneae: Leptonetidae)
Source: Sci Rep. 2022 Sep 15;12:15528. doi: 10.1038/s41598-022-18666-y (PMC9478141; doi:10.1038/s41598-022-18666-y)

[Supplementary Figure S1. IQ-tree of 424 COI sequences]

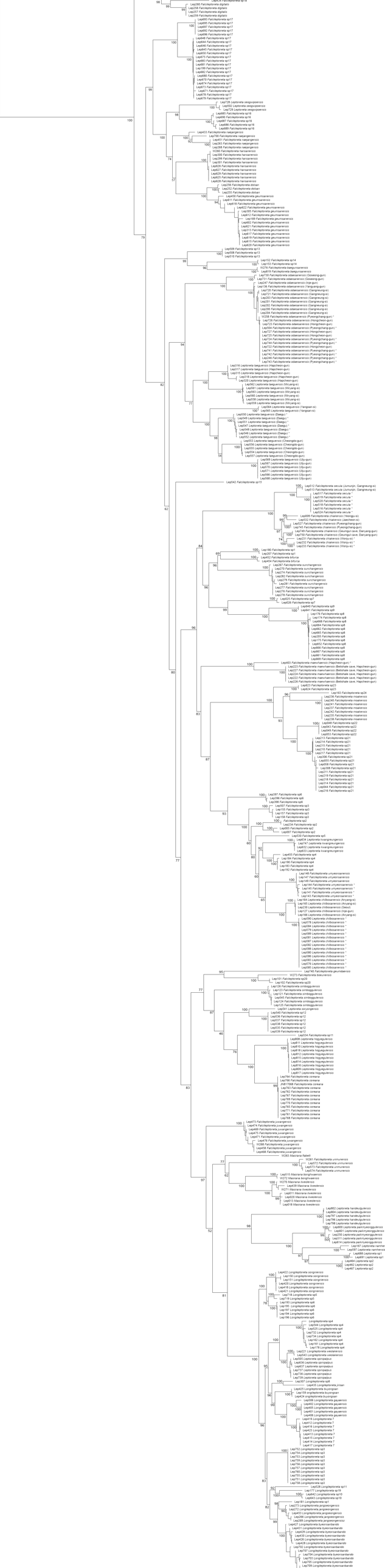

Supplement: Supplementary file 2 — Supplementary Information 2. [file 41598_2022_18666_MOESM2_ESM.pdf]
