# Supplementary material for: DNA barcodes reveal population-dependent cryptic diversity and various cases of sympatry of Korean leptonetid spiders (Araneae: Leptonetidae)
Source: Sci Rep. 2022 Sep 15;12:15528. doi: 10.1038/s41598-022-18666-y (PMC9478141; doi:10.1038/s41598-022-18666-y)
Supplement: Supplementary file 3 — Supplementary Information 3. [file 41598_2022_18666_MOESM3_ESM.pdf]

DNA barcodes reveal population-dependent cryptic diversity and various cases of sympatry of Korean leptonetid spiders (Araneae: Leptonetidae)

Jong-Hwa Oh[1], Sora Kim[3,4*] & Seunghwan Lee[1,2*]

[1]Laboratory of Insect Biosystematics, Department of Agricultural Biotechnology, Seoul National University

[2]Research Institute of Agriculture and Life Sciences, Seoul National University

[3]Laboratory of Insect Phylogenetics and Evolution, Department of Plant Protection & Quarantine, Jeonbuk National University

[4]Department of Agricultural Convergence Technology, Jeonbuk National University

*Correspondence and requests for materials should be addressed to S.K. (email: skim01@jbnu.ac.kr) or S.L. (email: seung@snu.ac.kr)

[Table S1. Collection data of 411 specimens with 13 NCBI data used in this study including Genbank Accession Numbers]

Voucher No.	Scientific name	Locality	GPS data										Collector	Collection Date	Genbank accession No.
Lep-005	*Falcileptoneta* sp2	Sucheol-ri, Punggi-eup, Yeongju-si, Gyeongsangbuk-do, Korea	36.91586111	36°	54’	57.1”	N	128°	27’	24.1”	128.4566944	E	J.W Kim & Y.G Choi	15.vi.2016	ON041801
Lep-007	*Falcileptoneta* sp2	Sucheol-ri, Punggi-eup, Yeongju-si, Gyeongsangbuk-do, Korea	36.91586111	36°	54’	57.1”	N	128°	27’	24.1”	128.4566944	E	J.W Kim & Y.G Choi	15.vi.2016	ON041802
Lep-009	*Falcileptoneta chiakensis*	Jwaseok-ri, Dansan-myeon, Yeongju-si, Gyeongsangbuk-do, Korea	36.99466667	36°	59’	40.8”	N	128°	34’	49.4”	128.5803889	E	J.W Kim & Y.G Choi	30.vii.2016	ON041803
Lep-011	*Masirana ilweolensis*	Jwaseok-ri, Dansan-myeon, Yeongju-si, Gyeongsangbuk-do, Korea	36.99466667	36°	59’	40.8”	N	128°	34’	49.4”	128.5803889	E	J.W Kim & Y.G Choi	30.vii.2016	ON041804
Lep-013	*Masirana ilweolensis*	Ojeon-ri, Mulya-myeon, Bonghwa-gun, Gyeongsangbuk-do, Korea	37.02438889	37°	01’	27.8”	N	128°	42’	25.8”	128.7071667	E	J.W Kim & Y.G Choi	30.vii.2016	ON041805
Lep-018	*Masirana ilweolensis*	661-29, Samga-ro, Punggi-eup, Yeongju-si, Gyeongsangbuk-do, Korea	36.93919444	36°	56’	21.1”	N	128°	30’	03.3”	128.5009167	E	J.W Kim & Y.G Choi	02.x.2016	ON041806
Lep-020	*Masirana ilweolensis*	661-29, Samga-ro, Punggi-eup, Yeongju-si, Gyeongsangbuk-do, Korea	36.93919444	36°	56’	21.1”	N	128°	30’	03.3”	128.5009167	E	J.W Kim & Y.G Choi	02.x.2016	ON041807
Lep-025	*Falcileptoneta* sp7	Bogildo Island: Jeongja-ri, Bogil-myeon, Wando-gun, Jeollanam-do, Korea	34.16458333	34°	09’	52.5”	N	126°	31’	08.4”	126.519	E	J.W Kim & Y.G Choi	29.iv.2017	ON041808
Lep-026	*Falcileptoneta* sp7	Bogildo Island: Jeongja-ri, Bogil-myeon, Wando-gun, Jeollanam-do, Korea	34.14005556	34°	08’	24.2”	N	126°	33’	46.6”	126.5629444	E	J.W Kim & Y.G Choi	30.iv.2017	ON041809
Lep-043	*Falcileptoneta* sp22	Deoksin-ri, Seolcheon-myeon, Namhae-gun, Gyeongsangnam-do, Korea	34.74063889	34°	44’	26.3”	N	127°	53’	34.6”	127.8929444	E	J.W Kim	06.viii.2014	ON041810
Lep-044	*Falcileptoneta* sp21	Deoksin-ri, Seolcheon-myeon, Namhae-gun, Gyeongsangnam-do, Korea	34.74063889	34°	44’	26.3”	N	127°	53’	34.6”	127.8929444	E	J.W Kim	06.viii.2014	ON041811
Lep-048	*Falcileptoneta* sp22	Deoksin-ri, Seolcheon-myeon, Namhae-gun, Gyeongsangnam-do, Korea	34.74063889	34°	44’	26.3”	N	127°	53’	34.6”	127.8929444	E	J.W Kim	06.viii.2014	ON041812
Lep-049	*Falcileptoneta* sp22	Deoksin-ri, Seolcheon-myeon, Namhae-gun, Gyeongsangnam-do, Korea	34.74063889	34°	44’	26.3”	N	127°	53’	34.6”	127.8929444	E	J.W Kim	06.viii.2014	ON041813
Lep-051	*Longileptoneta* sp7	Deoksin-ri, Seolcheon-myeon, Namhae-gun, Gyeongsangnam-do, Korea	34.74063889	34°	44’	26.3”	N	127°	53’	34.6”	127.8929444	E	J.W Kim & Y.G Choi	10.vi.2014	ON041814
Lep-053	*Falcileptoneta* sp22	Deoksin-ri, Seolcheon-myeon, Namhae-gun, Gyeongsangnam-do, Korea	34.74063889	34°	44’	26.3”	N	127°	53’	34.6”	127.8929444	E	J.W Kim & Y.G Choi	28.viii.2014	ON041814
Lep-055	*Falcileptoneta* sp21	Deoksin-ri, Seolcheon-myeon, Namhae-gun, Gyeongsangnam-do, Korea	34.74063889	34°	44’	26.3”	N	127°	53’	34.6”	127.8929444	E	J.W Kim & Y.G Choi	28.viii.2014	ON041816
Lep-058	*Falcileptoneta* sp21	Danghang-ri, Nam-myeon, Namhae-gun, Gyeongsangnam-do, Korea	34.78138889	34°	46’	53.0”	N	127°	53’	30.6”	127.8918333	E	J.W Kim & Y.G Choi	27.viii.2014	ON041817
Lep-101	*Falcileptoneta* sp20	Mt. Weolaksan: Mireuk-ri, Suanbo-myeon, Chungju-si, Chungcheongnam-do, Korea	36.82633333	36°	49’	34.8”	N	128°	04’	39.6”	128.0776667	E	J.W Kim & Y.G Choi	26.vi.2015	ON041818
Lep-102	*Falcileptoneta* sp20	Mt. Weolaksan: Mireuk-ri, Suanbo-myeon, Chungju-si, Chungcheongnam-do, Korea	36.82633333	36°	49’	34.8”	N	128°	04’	39.6”	128.0776667	E	J.W Kim & Y.G Choi	26.vi.2015	ON041819
Lep-121	*Falcileptoneta simboggulensis*	Simboggul Cave: Galgeum-ri, Yeonpung-myeon, Goesan-gun, Chungcheongbuk-do, Korea	36.78675	36°	47’	12.3”	N	127°	57’	52.6”	127.9646111	E	Y.G Choi	16.iii.2014	ON041820
Lep-123	*Falcileptoneta simboggulensis*	Simboggul Cave: Galgeum-ri, Yeonpung-myeon, Goesan-gun, Chungcheongbuk-do, Korea	36.78675	36°	47’	12.3”	N	127°	57’	52.6”	127.9646111	E	Y.G Choi	16.iii.2014	ON041821
Lep-124	*Falcileptoneta simboggulensis*	Simboggul Cave: Galgeum-ri, Yeonpung-myeon, Goesan-gun, Chungcheongbuk-do, Korea	36.78675	36°	47’	12.3”	N	127°	57’	52.6”	127.9646111	E	Y.G Choi	16.iii.2014	ON041822
Lep-125	*Falcileptoneta simboggulensis*	Simboggul Cave: Galgeum-ri, Yeonpung-myeon, Goesan-gun, Chungcheongbuk-do, Korea	36.78675	36°	47’	12.3”	N	127°	57’	52.6”	127.9646111	E	Y.G Choi	16.iii.2014	ON041823
Lep-126	*Falcileptoneta simboggulensis*	Simboggul Cave: Galgeum-ri, Yeonpung-myeon, Goesan-gun, Chungcheongbuk-do, Korea	36.78675	36°	47’	12.3”	N	127°	57’	52.6”	127.9646111	E	Y.G Choi	16.iii.2014	ON041824
Lep-127	*Leptoneta chilbosanensis*	Baekdamsa Temple, 746, Baekdam-ro, Buk-myeon, Inje-gun, Gangwon-do, Korea	38.16541667	38°	09’	55.5”	N	128°	22’	26.2”	128.3739444	E	J.H Oh	04.vi.2020-05.vi.2020	ON041825
Lep-136	*Falcileptoneta odaesanensis*	Inside valley, Osaek-ri, Seo-myeon, Yangyang-gun, Gangwon-do, Korea	38.075	38°	04’	30.0”	N	128°	28’	06.8”	128.4685556	E	J.S Oh	29.v.2020	ON041826
Lep-141	*Falcileptoneta umyeonsanensis*	Mt. Umyeonsan: 92, Gangnam-daero, 27-gil, Seocho-gu, Seoul, Korea	37.47897222	37°	28’	44.3”	N	127°	00’	53.3”	127.0314722	E	J.H Oh	14.vi.2020-16.vi.2020	ON041827
Lep-143	*Falcileptoneta umyeonsanensis*	Mt. Umyeon, San114, Umyeon-dong, Seocho-gu, Seoul, Korea	37.46275	37°	27’	45.9”	N	127°	00’	30.2”	127.0083889	E	J.H Oh	14.vi.2020-16.vi.2020	ON041828
Lep-144	*Falcileptoneta umyeonsanensis*	Mt. Umyeon, San114, Umyeon-dong, Seocho-gu, Seoul, Korea	37.46275	37°	27’	45.9”	N	127°	00’	30.2”	127.0083889	E	J.H Oh	14.vi.2020-16.vi.2020	ON041829
Lep-145	*Falcileptoneta umyeonsanensis*	Mt. Umyeon, San114, Umyeon-dong, Seocho-gu, Seoul, Korea	37.46275	37°	27’	45.9”	N	127°	00’	30.2”	127.0083889	E	J.H Oh	14.vi.2020-16.vi.2020	ON041830
Lep-147	*Falcileptoneta umyeonsanensis*	Mt. Kwanak, 193-1, Sillim-dong, Gwanak-gu, Seoul, Korea	37.45394444	37°	27’	14.2”	N	126°	56’	57.0”	126.9491667	E	J.H Oh	15.vi.2020-16.vi.2020	ON041831
Lep-148	*Falcileptoneta umyeonsanensis*	Mt. Kwanak, 193-1, Sillim-dong, Gwanak-gu, Seoul, Korea	37.45394444	37°	27’	14.2”	N	126°	56’	57.0”	126.9491667	E	J.H Oh	15.vi.2020-16.vi.2020	ON041832

Lep-149	*Falcileptoneta umyeonsanensis*	Mt. Kwanak, 193-1, Sillim-dong, Gwanak-gu, Seoul, Korea	37.45394444	37°	27'	14.2"	N	126°	56'	57.0"	126.9491667	E	J.H Oh	15.vi.2020-16.vi.2020	ON041833
Lep-150	*Longileptoneta songniensis*	Mt. Manisan: Sagi-ri, Hwado-myeon, Ganghwa-gun, Incheon, Korea	37.60647222	37°	36'	23.3"	N	126°	26'	43.6"	126.4454444	E	J.H Oh	20.vi.2020-21.vi.2020	ON041834
Lep-151	*Longileptoneta songniensis*	Mt. Manisan: Sagi-ri, Hwado-myeon, Ganghwa-gun, Incheon, Korea	37.6065	37°	36'	23.4"	N	126°	26'	42.2"	126.4450556	E	J.H Oh	20.vi.2020-21.vi.2020	ON041835
Lep-152	*Falcileptoneta* sp14	Mt. Gamaksan: 40-3, Gaekhyeon-ri, Jeokseong-myeon, Gyeonggi-do, Korea	37.94238889	37°	56'	32.6"	N	126°	58'	15.3"	126.9709167	E	J.H Oh	27.vi.2020-28.vi.2020	ON041836
Lep-153	*Falcileptoneta* sp14	Mt. Gamaksan: 40-3, Gaekhyeon-ri, Jeokseong-myeon, Gyeonggi-do, Korea	37.94155556	37°	56'	29.6"	N	126°	59'	52.9"	126.9980278	E	J.H Oh	27.vi.2020-28.vi.2020	ON041837
Lep-155	*Falcileptoneta* sp3	Mt. Sorisan: San42, Seoksan-ri, Danwol-myeon, Yangpyeong-gun, Gyeonggi-do, Korea	37.62966667	37°	37'	46.8"	N	127°	36'	34.5"	127.6095833	E	J.H Oh	11.vii.2020-12.vii.2020	ON041838
Lep-157	*Falcileptoneta* sp3	Mt. Sorisan: San42, Seoksan-ri, Danwol-myeon, Yangpyeong-gun, Gyeonggi-do, Korea	37.62966667	37°	37'	46.8"	N	127°	36'	34.5"	127.6095833	E	J.H Oh	11.vi.2020	ON041839
Lep-158	*Falcileptoneta* sp3	Mt. Sorisan: San42, Seoksan-ri, Danwol-myeon, Yangpyeong-gun, Gyeonggi-do, Korea	37.62966667	37°	37'	46.8"	N	127°	36'	34.5"	127.6095833	E	J.H Oh	12.vi.2020	ON041840
Lep-159	*Longileptoneta buyongsan*	Mt. Geodaesan: 145, Ijeonggol-ro, Sangdang-gu, Cheongju-si, Chungcheongbuk-do, Korea	36.64772222	36°	38'	51.8"	N	127°	31'	57.9"	127.53275	E	J.H Oh	16.vii.2020	ON041841
Lep-161	*Longileptoneta* sp3	Mt. Gyeryongsan: 145, Donghaksa 2-ro, Banpo-myeon, Gonju-si, Chungcheongnam-do, Korea	36.3575	36°	21'	27.0"	N	127°	14'	13.3"	127.2370278	E	J.H Oh	17.vii.2020-18.vii.2020	ON041842
Lep-162	*Longileptoneta* sp3	Mt. Gyeryongsan: 145, Donghaksa 2-ro, Banpo-myeon, Gonju-si, Chungcheongnam-do, Korea	36.3575	36°	21'	27.0"	N	127°	14'	13.3"	127.2370278	E	J.H Oh	17.vii.2020-18.vii.2020	ON041843
Lep-163	*Falcileptoneta* sp24	Mt. Gyeryongsan: 145, Donghaksa 2-ro, Banpo-myeon, Gonju-si, Chungcheongnam-do, Korea	36.3575	36°	21'	27.0"	N	127°	14'	13.3"	127.2370278	E	J.H Oh	17.vii.2020	ON041844
Lep-164	*Leptoneta chilbosanensis*	Mt. Surisan: 1150-4, Anyang-dong, Manan-gu, Anyang-si, Gyeonggi-do, Korea	37.37072222	37°	22'	14.6"	N	126°	54'	21.6"	126.906	E	J.H Oh	25.vii.2020-26.vii.2020	ON041845
Lep-165	*Leptoneta chilbosanensis*	Mt. Surisan: 1150-4, Anyang-dong, Manan-gu, Anyang-si, Gyeonggi-do, Korea	37.37072222	37°	22'	14.6"	N	126°	54'	21.6"	126.906	E	J.H Oh	25.vii.2020-26.vii.2020	ON041846
Lep-166	*Leptoneta chilbosanensis*	Mt. Surisan: 1150-4, Anyang-dong, Manan-gu, Anyang-si, Gyeonggi-do, Korea	37.37072222	37°	22'	14.6"	N	126°	54'	21.6"	126.906	E	J.H Oh	26.vii.2020	ON041847
Lep-167	*Leptoneta namhensis*	Heungguksa Temple: 21, Jungheung-dong, Yeosu-si, Jeollanam-do, Korea	34.82077778	34°	49'	14.8"	N	127°	41'	55.0"	127.6986111	E	J.H Oh	21.viii.2020-22.viii.2020	ON041848
Lep-169	*Falcileptoneta geumsanensis*	Heungguksa Temple: 21, Jungheung-dong, Yeosu-si, Jeollanam-do, Korea	34.82077778	34°	49'	14.8"	N	127°	41'	55.0"	127.6986111	E	J.H Oh	21.viii.2020-22.viii.2020	ON041849
Lep-174	*Falcileptoneta* sp8	Wando Arboretum: 156, Chopyeong 1-gil, Gunoe-myeon, Wando-gun, Jeollanam-do, Korea	34.36016667	34°	21'	36.6"	N	126°	39'	41.8"	126.6616111	E	J.H Oh	18.viii.2020-19.viii.2020	ON041850
Lep-175	*Falcileptoneta* sp8	Wando Arboretum: 156, Chopyeong 1-gil, Gunoe-myeon, Wando-gun, Jeollanam-do, Korea	34.36016667	34°	21'	36.6"	N	126°	39'	41.8"	126.6616111	E	J.H Oh	18.viii.2020-19.viii.2020	ON041851
Lep-176	*Falcileptoneta* sp8	Wando Arboretum: 156, Chopyeong 1-gil, Gunoe-myeon, Wando-gun, Jeollanam-do, Korea	34.36016667	34°	21'	36.6"	N	126°	39'	41.8"	126.6616111	E	J.H Oh	18.viii.2020-19.viii.2020	ON041852
Lep-177	*Longileptoneta* sp8	Wando Arboretum: 156, Chopyeong 1-gil, Gunoe-myeon, Wando-gun, Jeollanam-do, Korea	34.36016667	34°	21'	36.6"	N	126°	39'	41.8"	126.6616111	E	J.H Oh	18.viii.2020-19.viii.2020	ON041853
Lep-178	*Longileptoneta* sp3	Mt. Duryunsan: 1093, Hwangsan-ri, Hyeonsan-myeon, Haenam-gun, Jeollanam-do, Korea	34.45636111	34°	27'	22.9"	N	126°	35'	51.8"	126.5977222	E	J.H Oh	18.viii.2020-19.viii.2020	ON041854
Lep-180	*Falcileptoneta* sp1	Mt. Mudeungsan: 1555, Mudeung-ro, Buk-gu, Gwangju, Jeollanam-do, Korea	35.15077778	35°	09'	02.8"	N	126°	59'	21.7"	126.9893611	E	J.H Oh	15.viii.2020-17.viii.2020	ON041855
Lep-181	*Longileptoneta* sp1	Mt. Mudeungsan: 1555, Mudeung-ro, Buk-gu, Gwangju, Jeollanam-do, Korea	35.15077778	35°	09'	02.8"	N	126°	59'	21.7"	126.9893611	E	J.H Oh	15.viii.2020-17.viii.2020	ON041856
Lep-183	*Falcileptoneta* sp4	Valley, leaf litter, 854, Hangye-ri, Buk-myeon, Inje-gun, Gangwon-do, Korea	38.14630556	38°	08'	46.7"	N	128°	15'	50.0"	128.2638889	E	J.H Oh	24.viii.2020	ON041857
Lep-184	*Falcileptoneta* sp4	Valley, leaf litter, 854, Hangye-ri, Buk-myeon, Inje-gun, Gangwon-do, Korea	38.14630556	38°	08'	46.7"	N	128°	15'	50.0"	128.2638889	E	J.H Oh	24.viii.2020	ON041858
Lep-190	*Falcileptoneta* sp4	Valley, leaf litter, 854, Hangye-ri, Buk-myeon, Inje-gun, Gangwon-do, Korea	38.14630556	38°	08'	46.7"	N	128°	15'	50.0"	128.2638889	E	J.H Oh	24.viii.2020	ON041859
Lep-192	*Falcileptoneta* sp4	Valley, leaf litter, 854, Hangye-ri, Buk-myeon, Inje-gun, Gangwon-do, Korea	38.14630556	38°	08'	46.7"	N	128°	15'	50.0"	128.2638889	E	J.H Oh	24.viii.2020	ON041860
Lep-193	*Longileptoneta* sp5	Yonghyeon valley: 89-4, Yonghyeon-ri, Unsan-myeon, Seosan-si, Chungcheongnam-do, Korea	36.77541667	36°	46'	31.5"	N	126°	36'	16.1"	126.6044722	E	J.H Oh	19.ix.2020-20.ix.2020	ON041861
Lep-194	*Longileptoneta* sp5	Yonghyeon valley: 89-4, Yonghyeon-ri, Unsan-myeon, Seosan-si, Chungcheongnam-do, Korea	36.77533333	36°	46'	31.2"	N	126°	36'	14.3"	126.6039722	E	J.H Oh	20.ix.2020	ON041862
Lep-195	*Longileptoneta* sp5	Yonghyeon valley: 89-4, Yonghyeon-ri, Unsan-myeon, Seosan-si, Chungcheongnam-do, Korea	36.77533333	36°	46'	31.2"	N	126°	36'	14.3"	126.6039722	E	J.H Oh	20.ix.2020	ON041863
Lep-196	*Longileptoneta* sp5	Yonghyeon valley: 89-4, Yonghyeon-ri, Unsan-myeon, Seosan-si, Chungcheongnam-do, Korea	36.77533333	36°	46'	31.2"	N	126°	36'	14.3"	126.6039722	E	J.H Oh	20.ix.2020	ON041864
Lep-197	*Longileptoneta* sp5	Yonghyeon valley: 89-4, Yonghyeon-ri, Unsan-myeon, Seosan-si, Chungcheongnam-do, Korea	36.77533333	36°	46'	31.2"	N	126°	36'	14.3"	126.6039722	E	J.H Oh	20.ix.2020	ON041865
Lep-199	*Falcileptoneta* sp17	Wando Arboretum: 156, Chopyeong 1-gil, Gunoe-myeon, Wando-gun, Jeollanam-do, Korea	34.36016667	34°	21'	36.6"	N	126°	39'	41.8"	126.6616111	E	J.H Oh	01.x.2020	ON041866
Lep-200	*Falcileptoneta odaesanensis*	867, Seokgyo-ri, Sancheon-myeon, Gangneung-si, Gangwon-do, Korea	37.82433333	37°	49'	27.6"	N	128°	48'	03.5"	128.8009722	E	J.H Oh	03.x.2020	ON041867
Lep-201	*Falcileptoneta odaesanensis*	867, Seokgyo-ri, Sancheon-myeon, Gangneung-si, Gangwon-do, Korea	37.82433333	37°	49'	27.6"	N	128°	48'	03.5"	128.8009722	E	J.H Oh	04.x.2020	ON041868
Lep-202	*Falcileptoneta odaesanensis*	867, Seokgyo-ri, Sancheon-myeon, Gangneung-si, Gangwon-do, Korea	37.82433333	37°	49'	27.6"	N	128°	48'	03.5"	128.8009722	E	J.H Oh	04.x.2020	ON041869
Lep-203	*Falcileptoneta odaesanensis*	867, Seokgyo-ri, Sancheon-myeon, Gangneung-si, Gangwon-do, Korea	37.82433333	37°	49'	27.6"	N	128°	48'	03.5"	128.8009722	E	J.H Oh	04.x.2020	ON041870
Lep-204	*Falcileptoneta odaesanensis*	867, Seokgyo-ri, Sancheon-myeon, Gangneung-si, Gangwon-do, Korea	37.82433333	37°	49'	27.6"	N	128°	48'	03.5"	128.8009722	E	J.H Oh	04.x.2020	ON041871
Lep-205	*Falcileptoneta* sp8	Wando Arboretum: 156, Chopyeong 1-gil, Gunoe-myeon, Wando-gun, Jeollanam-do, Korea	34.36016667	34°	21'	36.6"	N	126°	39'	41.8"	126.6616111	E	J.H Oh	09.x.2020	ON041872
Lep-207	*Falcileptoneta* sp1	Mt. Mudeungsan: 1555, Mudeung-ro, Buk-gu, Gwangju, Jeollanam-do, Korea	35.15075	35°	09'	02.7"	N	126°	59'	23.6"	126.9898889	E	J.H Oh	01.x.2020-09.x.2020	ON041873
Lep-210	*Falcileptoneta* sp21	Namsan Park: 386, Dong-oe-ri, Goseong-eup, Gyeongsangnam-do, Korea	34.97013889	34°	58'	12.5"	N	128°	19'	41.3"	128.3281389	E	J.H Ahn	03.iv.2020	ON041874
Lep-211	*Falcileptoneta* sp21	Namsan Park: 386, Dong-oe-ri, Goseong-eup, Gyeongsangnam-do, Korea	34.97013889	34°	58'	12.5"	N	128°	19'	41.3"	128.3281389	E	J.H Ahn	03.iv.2020	ON041875
Lep-213	*Falcileptoneta* sp21	Namsan Park: 386, Dong-oe-ri, Goseong-eup, Gyeongsangnam-do, Korea	34.97013889	34°	58'	12.5"	N	128°	19'	41.3"	128.3281389	E	J.H Ahn	03.iv.2020	ON041876
Lep-214	*Falcileptoneta* sp21	Namsan Park: 386, Dong-oe-ri, Goseong-eup, Gyeongsangnam-do, Korea	34.97013889	34°	58'	12.5"	N	128°	19'	41.3"	128.3281389	E	J.H Ahn	03.iv.2020	ON041877
Lep-215	*Falcileptoneta* sp21	Namsan Park: 386, Dong-oe-ri, Goseong-eup, Gyeongsangnam-do, Korea	34.97013889	34°	58'	12.5"	N	128°	19'	41.3"	128.3281389	E	J.H Ahn	03.iv.2020	ON041878
Lep-216	*Falcileptoneta* sp21	Namsan Park: 386, Dong-oe-ri, Goseong-eup, Gyeongsangnam-do, Korea	34.97013889	34°	58'	12.5"	N	128°	19'	41.3"	128.3281389	E	J.H Ahn	03.iv.2020	ON041879
Lep-217	*Falcileptoneta* sp21	Namsan Park: 386, Dong-oe-ri, Goseong-eup, Gyeongsangnam-do, Korea	34.97013889	34°	58'	12.5"	N	128°	19'	41.3"	128.3281389	E	J.H Ahn	03.iv.2020	ON041880
Lep-218	*Falcileptoneta* sp21	Namsan Park: 386, Dong-oe-ri, Goseong-eup, Gyeongsangnam-do, Korea	34.97013889	34°	58'	12.5"	N	128°	19'	41.3"	128.3281389	E	J.H Ahn	03.iv.2020	ON041881
Lep-219	*Falcileptoneta* sp21	Namsan Park: 386, Dong-oe-ri, Goseong-eup, Gyeongsangnam-do, Korea	34.97013889	34°	58'	12.5"	N	128°	19'	41.3"	128.3281389	E	J.H Ahn	03.iv.2020	ON041882
Lep-221	*Longileptoneta weolakensis*	Mt. Baegunsan: Dopyeong-ri, Idong-myeon, Pocheon-si, Gyeonggi-do, Korea	38.07086111	38°	04'	15.1"	N	127°	24'	31.0"	127.4086111	E	S.W Yoon	24.viii.2020	ON041883
Lep-222	*Falcileptoneta maewhaensis*	Baetishale cave: San 117, Sayang-ri, Ssangchaek-myeon, Hapcheon-gun, Gyeongsangnam-do, Korea	35.61197222	35°	36'	43.1"	N	128°	15'	51.6"	128.2643333	E	J.H Oh	12.xii.2020	ON041884
Lep-223	*Falcileptoneta maewhaensis*	Baetishale cave: San 117, Sayang-ri, Ssangchaek-myeon, Hapcheon-gun, Gyeongsangnam-do, Korea	35.61197222	35°	36'	43.1"	N	128°	15'	51.6"	128.2643333	E	J.H Oh	12.xii.2020	ON041885
Lep-224	*Falcileptoneta maewhaensis*	Baetishale cave: San 117, Sayang-ri, Ssangchaek-myeon, Hapcheon-gun, Gyeongsangnam-do, Korea	35.61197222	35°	36'	43.1"	N	128°	15'	51.6"	128.2643333	E	J.H Oh	12.xii.2020	ON041886
Lep-226	*Falcileptoneta maewhaensis*	Baetishale cave: San 117, Sayang-ri, Ssangchaek-myeon, Hapcheon-gun, Gyeongsangnam-do, Korea	35.61197222	35°	36'	43.1"	N	128°	15'	51.6"	128.2643333	E	J.H Oh	12.xii.2020	ON041887
Lep-227	*Falcileptoneta maewhaensis*	Baetishale l cave: San 117, Sayang-ri, Ssangchaek-myeon, Hapcheon-gun, Gyeongsangnam-do, Korea	35.61197222	35°	36'	43.1"	N	128°	15'	51.6"	128.2643333	E	J.H Oh	12.xii.2020	ON041888
Lep-230	*Leptoneta chilbosanensis*	Mt. Kwanak, 193-1, Sillim-dong, Gwanak-gu, Seoul, Korea	37.45394444	37°	27'	14.2"	N	126°	56'	57.0"	126.9491667	E	J.H Oh & J.S Oh	15.x.2020	ON041889
Lep-231	*Falcileptoneta chiakensis*	Sajeong-ri, Eumseong-eup, Emseong-gun, Chungcheongbuk-do, Korea	N/A	-	-	-		-	-	-	N/A		Z. Zhao, S.T Kim	06.viii.2019	ON041890
Lep-232	*Falcileptoneta chiakensis*	Mt. Chiaksan: Hakgok-ri, Socho-myeon, Wonju-si, Gangwon-do, Korea	37.39922222	37°	23'	57.2"	N	128°	03'	03.1"	128.0508611	E	Z. Zhao, S.T Kim	17.ix.2018	ON041891
Lep-233	*Falcileptoneta chiakensis*	Mt. Chiaksan: Hakgok-ri, Socho-myeon, Wonju-si, Gangwon-do, Korea	37.39922222	37°	23'	57.2"	N	128°	03'	03.1"	128.0508611	E	Z. Zhao, S.T Kim	17.ix.2018	ON041892
Lep-234	*Falcileptoneta* sp2	Mt. Chiaksan: Hakgok-ri, Socho-myeon, Wonju-si, Gangwon-do, Korea	37.39922222	37°	23'	57.2"	N	128°	03'	03.1"	128.0508611	E	Z. Zhao, S.T Kim	17.ix.2018	ON041893
Lep-235	*Falcileptoneta moakensis*	Mt. Moaksan: Gui-myeon, Wanju-gun, Jeollabuk-do, Korea	35.72416667	35°	43'	27.0"	N	127°	03'	34.0"	127.0594444	E	Z. Zhao, S.T Kim	07.viii.2019	ON041894
Lep-236	*Falcileptoneta moakensis*	Mt. Moaksan: Gui-myeon, Wanju-gun, Jeollabuk-do, Korea	35.72416667	35°	43'	27.0"	N	127°	03'	34.0"	127.0594444	E	Z. Zhao, S.T Kim	07.viii.2019	ON041895
Lep-237	*Falcileptoneta moakensis*	Mt. Moaksan: Gui-myeon, Wanju-gun, Jeollabuk-do, Korea	35.72416667	35°	43'	27.0"	N	127°	03'	34.0"	127.0594444	E	Z. Zhao, S.T Kim	07.viii.2019	ON041896
Lep-238	*Falcileptoneta moakensis*	Mt. Moaksan: Gui-myeon, Wanju-gun, Jeollabuk-do, Korea	35.72416667	35°	43'	27.0"	N	127°	03'	34.0"	127.0594444	E	Z. Zhao, S.T Kim	07.viii.2019	ON041897
Lep-240	*Falcileptoneta moakensis*	Mt. Moaksan: Gui-myeon, Wanju-gun, Jeollabuk-do, Korea	35.72416667	35°	43'	27.0"	N	127°	03'	34.0"	127.0594444	E	Z. Zhao, S.T Kim	07.viii.2019	ON041898
Lep-241	*Falcileptoneta moakensis*	Mt. Moaksan: Gui-myeon, Wanju-gun, Jeollabuk-do, Korea	35.72416667	35°	43'	27.0"	N	127°	03'	34.0"	127.0594444	E	Z. Zhao, S.T Kim	07.viii.2019	ON041899
Lep-242	*Falcileptoneta moakensis*	Mt. Moaksan: Gui-myeon, , Wanju-gun, Jeollabuk-do, Korea	35.72416667	35°	43'	27.0"	N	127°	03'	34.0"	127.0594444	E	Z. Zhao, S.T Kim	07.viii.2019	ON041900
Lep-246	*Falcileptoneta odaesanensis*	Mt. Odaesan: Dongsan-ri, Jinbu-myeon, Pyeongchang-gun, Gangwon-do, Korea	37.7865	37°	47'	11.4"	N	128°	33'	59.4"	128.5665	E	Z. Zhao, S.T Kim	18.ix.2018	ON041901
Lep-247	*Falcileptoneta odaesanensis*	Mt. Seoraksan: Baekdam-ro, Buk-myeon, Inje-gun, Gangwon-do, Korea	N/A	-	-	-		-	-	-	N/A		Z. Zhao, S.T Kim	19.viii.2019	ON041902
Lep-250	*Leptoneta paikmyeonggulensis*	Seodeok-ri, Dolsan-eup, Yeosu-si, Jeollanam-do, Korea	34.64108333	34°	38'	27.9"	N	127°	45'	39.5"	127.7609722	E	Z. Zhao, S.T Kim	14.viii.2019	ON041903
Lep-252	*Falcileptoneta dolsan*	Seodeok-ri, Dolsan-eup, Yeosu-si, Jeollanam-do, Korea	34.64108333	34°	38'	27.9"	N	127°	45'	39.5"	127.7609722	E	Z. Zhao, S.T Kim	14.viii.2019	ON041904
Lep-255	*Falcileptoneta dolsan*	Seodeok-ri, Dolsan-eup, Yeosu-si, Jeollanam-do, Korea	34.64108333	34°	38'	27.9"	N	127°	45'	39.5"	127.7609722	E	Z. Zhao, S.T Kim	14.viii.2019	ON041905
Lep-256	*Falcileptoneta dolsan*	Seodeok-ri, Dolsan-eup, Yeosu-si, Jeollanam-do, Korea	34.64108333	34°	38'	27.9"	N	127°	45'	39.5"	127.7609722	E	Z. Zhao, S.T Kim	14.viii.2019	ON041906
Lep-257	*Falcileptoneta digitalis*	Mt. Jogyesan: Seokheung-ri, Nagan-myeon, Suncheon-si, Jeollanam-do, Korea	34.9625	34°	57'	45.0"	N	127°	20'	29.0"	127.3413889	E	Z. Zhao, S.T Kim	12.viii.2019	ON041907
Lep-258	*Falcileptoneta digitalis*	Mt. Jogyesan: Seokheung-ri, Nagan-myeon, Suncheon-si, Jeollanam-do, Korea	34.9625	34°	57'	45.0"	N	127°	20'	29.0"	127.3413889	E	Z. Zhao, S.T Kim	12.viii.2019	ON041908
Lep-259	*Falcileptoneta digitalis*	Mt. Jogyesan: Seokheung-ri, Nagan-myeon, Suncheon-si, Jeollanam-do, Korea	34.9625	34°	57'	45.0"	N	127°	20'	29.0"	127.3413889	E	Z. Zhao, S.T Kim	12.viii.2019	ON041909
Lep-260	*Falcileptoneta digitalis*	Mt. Jogyesan: Seokheung-ri, Nagan-myeon, Suncheon-si, Jeollanam-do, Korea	34.9625	34°	57'	45.0"	N	127°	20'	29.0"	127.3413889	E	Z. Zhao, S.T Kim	12.viii.2019	ON041910
Lep-263	*Falcileptoneta naejangensis*	Mt. Naejangsan: Bongdeok-ri, Bokheung-meyon, Sunchang-gun, Jeollabuk-do, Korea	35.46805556	35°	28'	05.0"	N	126°	52'	21.0"	126.8725	E	Z. Zhao, S.T Kim	09.viii.2019	ON041911
Lep-266	*Longileptoneta jangseongensis*	Mt. Naejangsan: Bongdeok-ri, Bokheung-meyon, Sunchang-gun, Jeollabuk-do, Korea	35.46805556	35°	28'	05.0"	N	126°	52'	21.0"	126.8725	E	Z. Zhao, S.T Kim	09.viii.2019	ON041912

Lep-267	*Falcileptoneta sunchangensis*	Mt. Naejangsan: Bongdeok-ri, Bokheung-meyon, Sunchang-gun, Jeollabuk-do, Korea	35.46805556	35°	28’	05.0”	N	126°	52’	21.0”	126.8725	E	Z. Zhao, S.T Kim	09.viii.2019	ON041913
Lep-268	*Falcileptoneta naejangensis*	Mt. Naejangsan: Bongdeok-ri, Bokheung-meyon, Sunchang-gun, Jeollabuk-do, Korea	35.46805556	35°	28’	05.0”	N	126°	52’	21.0”	126.8725	E	Z. Zhao, S.T Kim	09.viii.2019	ON041914
Lep-269	*Longileptoneta jangseongensis*	Mt. Naejangsan: Bongdeok-ri, Bokheung-meyon, Sunchang-gun, Jeollabuk-do, Korea	35.46805556	35°	28’	05.0”	N	126°	52’	21.0”	126.8725	E	Z. Zhao, S.T Kim	09.viii.2019	ON041915
Lep-270	*Falcileptoneta sunchangensis*	Mt. Naejangsan: Bongdeok-ri, Bokheung-meyon, Sunchang-gun, Jeollabuk-do, Korea	35.46805556	35°	28’	05.0”	N	126°	52’	21.0”	126.8725	E	Z. Zhao, S.T Kim	09.viii.2019	ON041916
Lep-272	*Longileptoneta jangseongensis*	Mt. Naejangsan: Bongdeok-ri, Bokheung-meyon, Sunchang-gun, Jeollabuk-do, Korea	35.46805556	35°	28’	05.0”	N	126°	52’	21.0”	126.8725	E	Z. Zhao, S.T Kim	09.viii.2019	ON041917
Lep-273	*Longileptoneta jangseongensis*	Mt. Naejangsan: Bongdeok-ri, Bokheung-meyon, Sunchang-gun, Jeollabuk-do, Korea	35.46805556	35°	28’	05.0”	N	126°	52’	21.0”	126.8725	E	Z. Zhao, S.T Kim	09.viii.2019	ON041918
Lep-274	*Falcileptoneta sunchangensis*	Mt. Naejangsan: Bongdeok-ri, Bokheung-meyon, Sunchang-gun, Jeollabuk-do, Korea	35.46805556	35°	28’	05.0”	N	126°	52’	21.0”	126.8725	E	Z. Zhao, S.T Kim	09.viii.2019	ON041919
Lep-276	*Falcileptoneta sunchangensis*	Mt. Naejangsan: Bongdeok-ri, Bokheung-meyon, Sunchang-gun, Jeollabuk-do, Korea	35.46805556	35°	28’	05.0”	N	126°	52’	21.0”	126.8725	E	Z. Zhao, S.T Kim	09.viii.2019	ON041920
Lep-277	*Falcileptoneta sunchangensis*	Mt. Naejangsan: Bongdeok-ri, Bokheung-meyon, Sunchang-gun, Jeollabuk-do, Korea	35.46805556	35°	28’	05.0”	N	126°	52’	21.0”	126.8725	E	Z. Zhao, S.T Kim	09.viii.2019	ON041921
Lep-278	*Falcileptoneta sunchangensis*	Mt. Naejangsan: Bongdeok-ri, Bokheung-meyon, Sunchang-gun, Jeollabuk-do, Korea	35.46805556	35°	28’	05.0”	N	126°	52’	21.0”	126.8725	E	Z. Zhao, S.T Kim	09.viii.2019	ON041922
Lep-279	*Falcileptoneta sunchangensis*	Mt. Naejangsan: Bongdeok-ri, Bokheung-meyon, Sunchang-gun, Jeollabuk-do, Korea	35.46805556	35°	28’	05.0”	N	126°	52’	21.0”	126.8725	E	Z. Zhao, S.T Kim	09.viii.2019	ON041923
Lep-281	*Falcileptoneta sunchangensis*	Mt. Naejangsan: Bongdeok-ri, Bokheung-meyon, Sunchang-gun, Jeollabuk-do, Korea	35.46805556	35°	28’	05.0”	N	126°	52’	21.0”	126.8725	E	Z. Zhao, S.T Kim	09.viii.2019	ON041924
Lep-282	*Falcileptoneta sunchangensis*	Mt. Naejangsan: Bongdeok-ri, Bokheung-meyon, Sunchang-gun, Jeollabuk-do, Korea	35.46805556	35°	28’	05.0”	N	126°	52’	21.0”	126.8725	E	Z. Zhao, S.T Kim	09.viii.2019	ON041925
Lep-299	*Falcileptoneta hansanensis*	Changjwa-ri, Hansan-myeon, Tongyeong-si, Gyeongsangnam-do, Korea	34.79611111	34°	47’	46.0”	N	128°	28’	40.0”	128.4777778	E	Z. Zhao, S.T Kim	22.ix.2018	ON041926
Lep-300	*Falcileptoneta hansanensis*	Changjwa-ri, Hansan-myeon, Tongyeong-si, Gyeongsangnam-do, Korea	34.79611111	34°	47’	46.0”	N	128°	28’	40.0”	128.4777778	E	Z. Zhao, S.T Kim	22.ix.2018	ON041927
Lep-301	*Falcileptoneta hansanensis*	Changjwa-ri, Hansan-myeon, Tongyeong-si, Gyeongsangnam-do, Korea	34.79611111	34°	47’	46.0”	N	128°	28’	40.0”	128.4777778	E	Z. Zhao, S.T Kim	22.ix.2018	ON041928
Lep-305	*Falcileptoneta geumsanensis*	Mt. Geumsan: Sangju-ri, Sangju-myeon, Namhae-gun, Gyeongsangnam-do, Korea	N/A	-	-	-		-	-	-	N/A		Z. Zhao, S.T Kim	14.viii.2019	ON041929
Lep-306	*Falcileptoneta* sp21	Mt. Geumsan: Sangju-ri, Sangju-myeon, Namhae-gun, Gyeongsangnam-do, Korea	N/A	-	-	-		-	-	-	N/A		Z. Zhao, S.T Kim	14.viii.2019	ON041930
Lep-308	*Falcileptoneta* sp21	Mt. Geumsan: Sangju-ri, Sangju-myeon, Namhae-gun, Gyeongsangnam-do, Korea	N/A	-	-	-		-	-	-	N/A		Z. Zhao, S.T Kim	14.viii.2019	ON041931
Lep-311	*Leptoneta paikmyeonggulensis*	Mt. Geumsan: Sangju-ri, Sangju-myeon, Namhae-gun, Gyeongsangnam-do, Korea	N/A	-	-	-		-	-	-	N/A		Z. Zhao, S.T Kim	14.viii.2019	ON041932
Lep-313	*Falcileptoneta geumsanensis*	Mt. Geumsan: Sangju-ri, Sangju-myeon, Namhae-gun, Gyeongsangnam-do, Korea	N/A	-	-	-		-	-	-	N/A		Z. Zhao, S.T Kim	14.viii.2019	ON041933
Lep-314	*Falcileptoneta* sp21	Mt. Geumsan: Sangju-ri, Sangju-myeon, Namhae-gun, Gyeongsangnam-do, Korea	N/A	-	-	-		-	-	-	N/A		Z. Zhao, S.T Kim	14.viii.2019	ON041934
Lep-315	*Leptoneta taeguensis*	Singye-ri, Seongju-gun, Gacheon-myeon, Gayasan, Gyeongsangbuk-do, Korea	35.83472222	35°	50’	05.0”	N	128°	10’	15.0”	128.1708333	E	Z. Zhao, S.T Kim	16.viiii.2019	ON041935
Lep-316	*Leptoneta taeguensis*	Singye-ri, Seongju-gun, Gacheon-myeon, Gayasan, Gyeongsangbuk-do, Korea	35.83472222	35°	50’	05.0”	N	128°	10’	15.0”	128.1708333	E	Z. Zhao, S.T Kim	16.viiii.2019	ON041936
Lep-317	*Leptoneta taeguensis*	Singye-ri, Seongju-gun, Gacheon-myeon, Gayasan, Gyeongsangbuk-do, Korea	35.83472222	35°	50’	05.0”	N	128°	10’	15.0”	128.1708333	E	Z. Zhao, S.T Kim	16.viiii.2019	ON041937
Lep-318	*Leptoneta taeguensis*	Singye-ri, Seongju-gun, Gacheon-myeon, Gayasan, Gyeongsangbuk-do, Korea	35.83472222	35°	50’	05.0”	N	128°	10’	15.0”	128.1708333	E	Z. Zhao, S.T Kim	16.viiii.2019	ON041938
Lep-320	*Leptoneta taeguensis*	Singye-ri, Seongju-gun, Gacheon-myeon, Gayasan, Gyeongsangbuk-do, Korea	35.83472222	35°	50’	05.0”	N	128°	10’	15.0”	128.1708333	E	Z. Zhao, S.T Kim	16.viiii.2019	ON041939
Lep-396	*Falcileptoneta* sp6	Mt. Bongjaesan, Boodaegul cave: Donchun-dong, Yeonsu-gu, Incheon, Korea	37.937767	37°	23’	52.0”	N	126°	39’	49.4”	126.663707	E	Y.G Choi	09.vi.2008	ON041940
Lep-397	*Falcileptoneta* sp6	Mt. Bongjaesan, Boodaegul cave: Donchun-dong, Yeonsu-gu, Incheon, Korea	37.937767	37°	23’	52.0”	N	126°	39’	49.4”	126.663707	E	Y.G Choi	09.vi.2008	ON041941
Lep-398	*Falcileptoneta* sp6	Mt. Bongjaesan, Boodaegul cave: Donchun-dong, Yeonsu-gu, Incheon, Korea	37.937767	37°	23’	52.0”	N	126°	39’	49.4”	126.663707	E	Y.G Choi	09.vi.2008	ON041942
Lep-401	*Longileptoneta gayaensis*	Mt. Gayasan: Sinpa-ri, Suryun-myeon, Seongju-gun, Gyeongsangbuk-do, Korea	35.82	35°	49’	12.0”	N	128°	10’	48.0”	128.18	E	Z. Zhao, S.T Kim	25.ix.2018	ON041943
Lep-402	*Longileptoneta gayaensis*	Mt. Gayasan: Sinpa-ri, Suryun-myeon, Seongju-gun, Gyeongsangbuk-do, Korea	35.82	35°	49’	12.0”	N	128°	10’	48.0”	128.18	E	Z. Zhao, S.T Kim	30.x.2018	ON041944
Lep-403	*Falcileptoneta maewhaensis*	Mt. Gayasan: Sinpa-ri, Suryun-myeon, Seongju-gun, Gyeongsangbuk-do, Korea	35.82	35°	49’	12.0”	N	128°	10’	48.0”	128.18	E	Z. Zhao, S.T Kim	30.x.2018	ON041945
Lep-405	*Longileptoneta gayaensis*	Mt. Gayasan: Sinpa-ri, Suryun-myeon, Seongju-gun, Gyeongsangbuk-do, Korea	35.82	35°	49’	12.0”	N	128°	10’	48.0”	128.18	E	Z. Zhao, S.T Kim	30.x.2018	ON041946
Lep-406	*Longileptoneta gayaensis*	Mt. Gayasan: Sinpa-ri, Suryun-myeon, Seongju-gun, Gyeongsangbuk-do, Korea	35.82	35°	49’	12.0”	N	128°	10’	48.0”	128.18	E	Z. Zhao, S.T Kim	30.x.2018	ON041947
Lep-412	*Longileptoneta* sp6	Mt. Songnisan: Donggwan-ri, Hwanam-myeon, Sangju-si, Gyeongsangbuk-do, Korea	36.51305556	36°	30’	47.0”	N	127°	55’	21.0”	127.9225	E	Z. Zhao, S.T Kim	30.x.2018	ON041948
Lep-413	*Longileptoneta* sp6	Mt. Songnisan: Donggwan-ri, Hwanam-myeon, Sangju-si, Gyeongsangbuk-do, Korea	36.51305556	36°	30’	47.0”	N	127°	55’	21.0”	127.9225	E	Z. Zhao, S.T Kim	30.x.2018	ON041949
Lep-414	*Longileptoneta* sp6	Mt. Songnisan: Donggwan-ri, Hwanam-myeon, Sangju-si, Gyeongsangbuk-do, Korea	36.51305556	36°	30’	47.0”	N	127°	55’	21.0”	127.9225	E	Z. Zhao, S.T Kim	30.x.2018	ON041950
Lep-415	*Longileptoneta* sp6	Mt. Songnisan: Donggwan-ri, Hwanam-myeon, Sangju-si, Gyeongsangbuk-do, Korea	36.51305556	36°	30’	47.0”	N	127°	55’	21.0”	127.9225	E	Z. Zhao, S.T Kim	30.x.2018	ON041951
Lep-416	*Longileptoneta* sp6	Mt. Songnisan: Donggwan-ri, Hwanam-myeon, Sangju-si, Gyeongsangbuk-do, Korea	36.51305556	36°	30’	47.0”	N	127°	55’	21.0”	127.9225	E	Z. Zhao, S.T Kim	30.x.2018	ON041952
Lep-417	*Longileptoneta* sp6	Mt. Songnisan: Donggwan-ri, Hwanam-myeon, Sangju-si, Gyeongsangbuk-do, Korea	36.51305556	36°	30’	47.0”	N	127°	55’	21.0”	127.9225	E	Z. Zhao, S.T Kim	30.x.2018	ON041953
Lep-418	*Longileptoneta songniensis*	Mt. Songnisan: Donggwan-ri, Hwanam-myeon, Sangju-si, Gyeongsangbuk-do, Korea	36.51305556	36°	30’	47.0”	N	127°	55’	21.0”	127.9225	E	Z. Zhao, S.T Kim	30.x.2018	ON041954
Lep-419	*Longileptoneta* sp6	Mt. Songnisan: Donggwan-ri, Hwanam-myeon, Sangju-si, Gyeongsangbuk-do, Korea	36.51305556	36°	30’	47.0”	N	127°	55’	21.0”	127.9225	E	Z. Zhao, S.T Kim	30.x.2018	ON041955
Lep-420	*Longileptoneta songniensis*	Mt. Songnisan: Donggwan-ri, Hwanam-myeon, Sangju-si, Gyeongsangbuk-do, Korea	36.51305556	36°	30’	47.0”	N	127°	55’	21.0”	127.9225	E	Z. Zhao, S.T Kim	30.x.2018	ON041956
Lep-421	*Longileptoneta songniensis*	Mt. Songnisan: Donggwan-ri, Hwanam-myeon, Sangju-si, Gyeongsangbuk-do, Korea	36.51305556	36°	30’	47.0”	N	127°	55’	21.0”	127.9225	E	Z. Zhao, S.T Kim	30.x.2018	ON041957
Lep-422	*Longileptoneta songniensis*	Mt. Songnisan: Donggwan-ri, Hwanam-myeon, Sangju-si, Gyeongsangbuk-do, Korea	36.51305556	36°	30’	47.0”	N	127°	55’	21.0”	127.9225	E	Z. Zhao, S.T Kim	30.x.2018	ON041958
Lep-423	*Longileptoneta* sp6	Mt. Songnisan: Donggwan-ri, Hwanam-myeon, Sangju-si, Gyeongsangbuk-do, Korea	36.51305556	36°	30’	47.0”	N	127°	55’	21.0”	127.9225	E	Z. Zhao, S.T Kim	30.x.2018	ON041959
Lep-424	*Longileptoneta buyongsan*	Eumseong-gun, Eumseong-eup, Sajeong-ri, Chungcheongbuk-do, Korea	36.97025	36°	58’	12.9”	N	127°	37’	10.6”	127.6196111	E	Z. Zhao, S.T Kim	06.viii.2019	ON041960
Lep-425	*Longileptoneta buyongsan*	Eumseong-gun, Eumseong-eup, Sajeong-ri, Chungcheongbuk-do, Korea	36.97025	36°	58’	12.9”	N	127°	37’	10.6”	127.6196111	E	Z. Zhao, S.T Kim	06.viii.2019	ON041961
Lep-426	*Longileptoneta byeonsanbando*	Jangsin-ri, Haseo-myeon, Buan-gun, Jeollabuk-do, Korea	35.670146	35°	40’	12.5”	N	126°	37’	45.3”	126.629253	E	Z. Zhao, S.T Kim	08.viii.2019	ON041962
Lep-427	*Longileptoneta byeonsanbando*	Jangsin-ri, Haseo-myeon, Buan-gun, Jeollabuk-do, Korea	35.670146	35°	40’	12.5”	N	126°	37’	45.3”	126.629253	E	Z. Zhao, S.T Kim	08.viii.2019	ON041963
Lep-428	*Longileptoneta byeonsanbando*	Jangsin-ri, Haseo-myeon, Buan-gun, Jeollabuk-do, Korea	35.670146	35°	40’	12.5”	N	126°	37’	45.3”	126.629253	E	Z. Zhao, S.T Kim	08.viii.2019	ON041964
Lep-429	*Longileptoneta byeonsanbando*	Jangsin-ri, Haseo-myeon, Buan-gun, Jeollabuk-do, Korea	35.670146	35°	40’	12.5”	N	126°	37’	45.3”	126.629253	E	Z. Zhao, S.T Kim	08.viii.2019	ON041965
Lep-430	*Longileptoneta byeonsanbando*	Jangsin-ri, Haseo-myeon, Buan-gun, Jeollabuk-do, Korea	35.670146	35°	40’	12.5”	N	126°	37’	45.3”	126.629253	E	Z. Zhao, S.T Kim	08.viii.2019	ON041966
Lep-431	*Longileptoneta byeonsanbando*	Jangsin-ri, Haseo-myeon, Buan-gun, Jeollabuk-do, Korea	35.670146	35°	40’	12.5”	N	126°	37’	45.3”	126.629253	E	Z. Zhao, S.T Kim	08.viii.2019	ON041967
Lep-433	*Falcileptoneta naejangensis*	Mt. Jirisan: Daeseong-ri, Hwagae-myeon, Hadong-gun, Gyeongsangnam-do, Korea	35.27397222	35°	16’	26.3”	N	127°	39’	26.8”	127.6574444	E	Z. Zhao, S.T Kim	15.viii.2019	ON041968
Lep-434	*Falcileptoneta* sp18	Mt. Jirisan: Daeseong-ri, Hwagae-myeon, Hadong-gun, Gyeongsangnam-do, Korea	35.27397222	35°	16’	26.3”	N	127°	39’	26.8”	127.6574444	E	Z. Zhao, S.T Kim	15.viii.2019	ON041969
Lep-435	*Longileptoneta jirisan*	Mt. Jirisan: Daeseong-ri, Hwagae-myeon, Hadong-gun, Gyeongsangnam-do, Korea	35.27397222	35°	16’	26.3”	N	127°	39’	26.8”	127.6574444	E	Z. Zhao, S.T Kim	15.viii.2019	ON041970
Lep-439	*Masirana ilweolensis*	Mt. Irwolsan: Dang-ri, Cheonggi-myeon, Yeongyang-gun, Gyeongsangbuk-do, Korea	N/A	-	-	-		-	-	-	N/A		Z. Zhao, S.T Kim	30.x.2018	ON041971
Lep-450	*Falcileptoneta geumsanensis*	Sunchang-gun, Bokheung-myeon, Bongdeok-ri, Naejangsan	N/A	-	-	-		-	-	-	N/A		Z. Zhao, S.T Kim	09.viii.2019	ON041972
Lep-451	*Falcileptoneta naejanensis*	Sunchang-gun, Bokheung-myeon, Bongdeok-ri, Naejangsan	N/A	-	-	-		-	-	-	N/A		Z. Zhao, S.T Kim	09.viii.2019	ON041973
Lep-452	*Falcileptoneta bifurca*	Mt. Naejangsan: Sinseong-ri, Bukha-myeon, Jangseong-gun, Jeollanam-do, Korea	35.4475	35°	26’	51.0”	N	126°	50’	44.0”	126.8455556	E	Z. Zhao, S.T Kim	09.viii.2019	ON041974
Lep-453	*Longileptoneta jangseongensis*	Mt. Naejangsan: Sinseong-ri, Bukha-myeon, Jangseong-gun, Jeollanam-do, Korea	35.4475	35°	26’	51.0”	N	126°	50’	44.0”	126.8455556	E	Z. Zhao, S.T Kim	09.viii.2019	ON041975
Lep-454	*Falcileptoneta bifurca*	Mt. Naejangsan: Sinseong-ri, Bukha-myeon, Jangseong-gun, Jeollanam-do, Korea	35.4475	35°	26’	51.0”	N	126°	50’	44.0”	126.8455556	E	Z. Zhao, S.T Kim	09.viii.2019	ON041976
Lep-455	*Falcileptoneta* sp4	Mt. Seoraksan: Baekdam-ro, Buk-myeon, Inje-gun, Gangwon-do, Korea	N/A	-	-	-		-	-	-	N/A		Z. Zhao, S.T Kim	19.viii.2019	ON041977
Lep-458	*Falcileptoneta juwangensis*	Mujanggul-cave, Mt. Juwangsan: Sangeui-ri, Juwangsan-myeon, Cheongsong-gun, Gyeongsangbuk-do, Korea	36.39522222	36°	23’	42.8”	N	129°	09’	25.0”	129.1569444	E	Z. Zhao, S.T Kim	30.x.2018	ON041978
Lep-462	*Leptoneta* sp2	Mt. Wolchulsan: Dogap-ri, Gumseo-myeon, Yongam-gun, Jeollanam-do, Korea	34.75316667	34°	45’	11.4”	N	126°	39’	53.4”	126.6648333	E	Z. Zhao, S.T Kim	10.viii.2019	ON041979
Lep-464	*Leptoneta* sp2	Mt. Wolchulsan: Dogap-ri, Gumseo-myeon, Yongam-gun, Jeollanam-do, Korea	34.75316667	34°	45’	11.4”	N	126°	39’	53.4”	126.6648333	E	Z. Zhao, S.T Kim	10.viii.2019	ON041980
Lep-467	*Leptoneta* sp2	Mt. Wolchulsan: Dogap-ri, Gumseo-myeon, Yongam-gun, Jeollanam-do, Korea	34.75316667	34°	45’	11.4”	N	126°	39’	53.4”	126.6648333	E	Z. Zhao, S.T Kim	10.viii.2019	ON041981
Lep-468	*Falcileptoneta juwangensis*	Mujanggul-cave, Mt. Juwangsan: Sangeui-ri, Juwangsan-myeon, Cheongsong-gun, Gyeongsangbuk-do, Korea	36.39522222	36°	23’	42.8”	N	129°	09’	25.0”	129.1569444	E	Z. Zhao, S.T Kim	30.x.2018	ON041982
Lep-469	*Falcileptoneta juwangensis*	Mt. Juwangsan: Sangeui-ri, Juwangsan-myeon, Cheongsong-gun, Gyeongsangbuk-do, Korea	36.39933333	36°	23’	57.6”	N	129°	09’	17.3”	129.1548056	E	Z. Zhao, S.T Kim	27.ix.2018	ON041983
Lep-471	*Falcileptoneta juwangensis*	Mt. Juwangsan: Sangeui-ri, Juwangsan-myeon, Cheongsong-gun, Gyeongsangbuk-do, Korea	36.39933333	36°	23’	57.6”	N	129°	09’	17.3”	129.1548056	E	Z. Zhao, S.T Kim	27.ix.2018	ON041984
Lep-473	*Falcileptoneta juwangensis*	Mt. Juwangsan: Sangeui-ri, Juwangsan-myeon, Cheongsong-gun, Gyeongsangbuk-do, Korea	36.39933333	36°	23’	57.6”	N	129°	09’	17.3”	129.1548056	E	Z. Zhao, S.T Kim	27.ix.2018	ON041985
Lep-474	*Falcileptoneta juwangensis*	Mt. Juwangsan: Sangeui-ri, Juwangsan-myeon, Cheongsong-gun, Gyeongsangbuk-do, Korea	36.39933333	36°	23’	57.6”	N	129°	09’	17.3”	129.1548056	E	Z. Zhao, S.T Kim	27.ix.2018	ON041986
Lep-475	*Falcileptoneta juwangensis*	Mt. Juwangsan: Sangeui-ri, Juwangsan-myeon, Cheongsong-gun, Gyeongsangbuk-do, Korea	36.39933333	36°	23’	57.6”	N	129°	09’	17.3”	129.1548056	E	Z. Zhao, S.T Kim	27.ix.2018	ON041987
Lep-476	*Falcileptoneta juwangensis*	Mt. Juwangsan: Sangeui-ri, Juwangsan-myeon, Cheongsong-gun, Gyeongsangbuk-do, Korea	36.39933333	36°	23’	57.6”	N	129°	09’	17.3”	129.1548056	E	Z. Zhao, S.T Kim	27.ix.2018	ON041988
Lep-502	*Leptoneta seogwipoensis*	1503, Sanghyo-dong, Seogwipo-si, Jeju, Korea	33.29997222	33°	17’	59.9”	N	126°	34’	54.6”	126.5818333	E	J.G Lee & J.H Lee	22.vi.2020	ON041989
Lep-504	*Falcileptoneta odesanensis*	Mt. Odaesan: Dongsan-ri, Jinbu-myeon, Pyeongchang-gun, Gangwon-do, Korea	37.78469444	37°	47’	04.9”	N	128°	34’	24.2”	128.5733889	E	J.G Lee & J.H Lee	14.vii.2020	ON041990
Lep-505	*Leptoneta spinipalpus*	Mt. Suraksan: Sanggye 1-dong, Nowon-gu, Seoul, Korea	37.69652778	37°	41’	47.5”	N	127°	04’	50.7”	127.08075	E	H.J Song	27.ii.2021	ON041991
Lep-506	*Longileptoneta gayaensis*	Outside Baetishale cave: San 117, Sayang-ri, Ssangchaek-myeon, Hapcheon-gun, Gyeongsangnam-do, Korea	35.61175	35°	36’	42.3”	N	128°	15’	53.7”	128.2649167	E	J.H Oh	17.iv.2021	ON041992

Lep-507	*Falcileptoneta* sp3	150-4, Misa-ri, Seorak-myeon, Gapyeong-gun, Gyeonggi-do, Korea	37.70172222	37°	42'	06.2"	N	127°	32'	40.5"	127.5445833	E	H.J Song	03.v.2021	ON041993
Lep-508	*Falcileptoneta* sp13	Gisan-ri, Gwangtan-myeon, Paju-si, Gyeonggi-do, Korea	37.767	37°	46'	01.2"	N	126°	55'	24.7"	126.9235278	E	J.W Seo	18.iv.2020	ON041994
Lep-509	*Falcileptoneta* sp13	Gisan-ri, Gwangtan-myeon, Paju-si, Gyeonggi-do, Korea	37.767	37°	46'	01.2"	N	126°	55'	24.7"	126.9235278	E	J.W Seo	18.iv.2020	ON041995
Lep-510	*Falcileptoneta* sp13	Gisan-ri, Gwangtan-myeon, Paju-si, Gyeonggi-do, Korea	37.767	37°	46'	01.2"	N	126°	55'	24.7"	126.9235278	E	J.W Seo	18.iv.2020	ON041996
Lep-512	*Falcileptoneta secula*	Samgyo-ri, Jumunjin-eup, Gangneung-si, Gangwon-do, Korea	37.87722222	37°	52'	38.0"	N	128°	40'	56.3"	128.6823056	E	J.W Seo	25.v.2020	ON041997
Lep-513	*Falcileptoneta secula*	Samgyo-ri, Jumunjin-eup, Gangneung-si, Gangwon-do, Korea	37.87722222	37°	52'	38.0"	N	128°	40'	56.3"	128.6823056	E	J.W Seo	25.v.2020	ON041998
Lep-515	*Masirana bonghwaensis*	159, Cheoram-dong, Taebaek-si, Gangwon-do, Korea	37.13711111	37°	08'	13.6"	N	129°	03'	29.8"	129.0582778	E	J.W Seo	05.v.2020	ON041999
Lep-516	*Falcileptoneta secula*	Magwihalmigul cave: Nakpung-ri, Okgye-myeon, Gangneung-si, Gangwon-do, Korea	37.62258333	37°	37'	21.3"	N	129°	00'	34.6"	129.0096111	E	J.H Oh	24.iv.2021	ON042000
Lep-517	*Falcileptoneta secula*	Magwihalmigul cave: Nakpung-ri, Okgye-myeon, Gangneung-si, Gangwon-do, Korea	37.62258333	37°	37'	21.3"	N	129°	00'	34.6"	129.0096111	E	J.H Oh	24.iv.2021	ON042001
Lep-518	*Falcileptoneta secula*	Magwihalmigul cave: Nakpung-ri, Okgye-myeon, Gangneung-si, Gangwon-do, Korea	37.62258333	37°	37'	21.3"	N	129°	00'	34.6"	129.0096111	E	J.H Oh	24.iv.2021	ON042002
Lep-519	*Falcileptoneta secula*	Magwihalmigul cave: Nakpung-ri, Okgye-myeon, Gangneung-si, Gangwon-do, Korea	37.62258333	37°	37'	21.3"	N	129°	00'	34.6"	129.0096111	E	J.H Oh	24.iv.2021	ON042003
Lep-520	*Falcileptoneta secula*	Magwihalmigul cave: Nakpung-ri, Okgye-myeon, Gangneung-si, Gangwon-do, Korea	37.62258333	37°	37'	21.3"	N	129°	00'	34.6"	129.0096111	E	J.H Oh	24.iv.2021	ON042004
Lep-524	*Falcileptoneta secula*	Magwihalmigul cave: Nakpung-ri, Okgye-myeon, Gangneung-si, Gangwon-do, Korea	37.62258333	37°	37'	21.3"	N	129°	00'	34.6"	129.0096111	E	J.H Oh	24.iv.2021	ON042005
Lep-525	*Longileptoneta* sp3	Gwangreung Aboretum: 415, Gwangneungsumogwon-ro, Soheul-eup, Pocheon-si, Gyeonggi-do, Korea	37.75905556	37°	45'	32.6"	N	127°	10'	02.1"	127.16725	E	J.H Oh & W.O Kim	03.v.2021	ON042006
Lep-527	*Falcileptoneta chiakensis*	Mt. Gariwangsan: 115-2, Jangjeon-ri, Jinbu-myeon, Pyeongchang-gun, Gangwon-do, Korea	37.49166667	37°	29'	30.0"	N	128°	32'	31.0"	128.5419444	E	J.H Oh	12.vi.2021	ON042007
Lep-528	*Longileptoneta* sp10	Mt. Amisan: Jukdong-ri, Myeoncheon-myeon, Dangjin-si, Chungcheongnam-do, Korea	36.84772222	36°	50'	51.8"	N	126°	39'	35.4"	126.6598333	E	J.H Oh	24.vi.2021-02.vii.2021	ON042008
Lep-530	*Falcileptoneta* sp5	Mt. Yeonginsan: Asan-ri, Yeongin-meyon, Asansi, Chungcheongnam-do, Korea	36.84516667	36°	50'	42.6"	N	126°	57'	13.7"	126.9538056	E	J.H Oh	24.vi.2021-02.vii.2021	ON042009
Lep-532	*Falcileptoneta chiakensis*	Mt. Weolaksan, Bodeok cave: 225, Hucheonggol-gil, Deoksan-myeon, Jecheon-si, Chungcheongbuk-do, Korea	36.89972222	36°	53'	59.0"	N	128°	05'	25.9"	128.0905278	E	J.H Oh	10.vii.2021	ON042010
Lep-534	*Falcileptoneta* sp11	Gongigul cave: Gongi-ri, Salmi-myeon, Chungju-si, Chungcheongbuk-do, Korea	36.89844444	36°	53'	54.4"	N	128°	03'	11.0"	128.0530556	E	J.H Oh	10.vii.2021	ON042011
Lep-535	*Falcileptoneta* sp12	Changseokgul cave, 12-16, Unam 3-gil, Miwon-myeon, Sangdang-gu, Chungju-si, Chungcheongbuk-do, Korea	36.61322222	36°	36'	47.6"	N	127°	40'	58.6"	127.6829444	E	J.H Oh	11.vii.2021	ON042012
Lep-536	*Falcileptoneta* sp12	Changseokgul cave, 12-16, Unam 3-gil, Miwon-myeon, Sangdang-gu, Chungju-si, Chungcheongbuk-do, Korea	36.61322222	36°	36'	47.6"	N	127°	40'	58.6"	127.6829444	E	J.H Oh	11.vii.2021	ON042013
Lep-537	*Falcileptoneta* sp12	Changseokgul cave, 12-16, Unam 3-gil, Miwon-myeon, Sangdang-gu, Chungju-si, Chungcheongbuk-do, Korea	36.61322222	36°	36'	47.6"	N	127°	40'	58.6"	127.6829444	E	J.H Oh	11.vii.2021	ON042014
Lep-538	*Falcileptoneta* sp12	Changseokgul cave, 12-16, Unam 3-gil, Miwon-myeon, Sangdang-gu, Chungju-si, Chungcheongbuk-do, Korea	36.61322222	36°	36'	47.6"	N	127°	40'	58.6"	127.6829444	E	J.H Oh	11.vii.2021	ON042015
Lep-539	*Falcileptoneta* sp12	Changseokgul cave, 12-16, Unam 3-gil, Miwon-myeon, Sangdang-gu, Chungju-si, Chungcheongbuk-do, Korea	36.61322222	36°	36'	47.6"	N	127°	40'	58.6"	127.6829444	E	J.H Oh	11.vii.2021	ON042016
Lep-540	*Falcileptoneta* sp12	Changseokgul cave, 12-16, Unam 3-gil, Miwon-myeon, Sangdang-gu, Chungju-si, Chungcheongbuk-do, Korea	36.61322222	36°	36'	47.6"	N	127°	40'	58.6"	127.6829444	E	J.H Oh	11.vii.2021	ON042017
Lep-541	*Leptoneta soryongensis*	Soryong cave, 35, Cheongnamdae-gil, Munui-myeon, Sangdang-gu, Chungju-si, Chungcheongbuk-do, Korea	36.50286111	36°	30'	10.3"	N	127°	31'	05.4"	127.5181667	E	J.H Oh	11.vii.2021	ON042018
Lep-542	*Falcileptoneta* sp15	Mt. Odaesan: Dongsan-ri, Jinbu-myeon, Pyeongchang-gun, Gangwon-do, Korea	37.78469444	37°	47'	04.9"	N	128°	34'	24.2"	128.5733889	E	J.W Seo	11.vii.2021	ON042019
Lep-543	*Longileptoneta weolakensis*	Mt. Weolaksan 205-1, Songgye-ri, Hansu-myeon, Jecheon-si, Chungcheongbuk-do, Korea	36.88161111	36°	52'	53.8"	N	128°	05'	05.3"	128.0848056	E	J.W Seo	11.vii.2021	ON042020
Lep-544	*Longileptoneta* sp3	Outside simboggul cave: Galgeum-ri, Yeonpung-myeon, Goesan-gun, Chungcheongbuk-do, Korea	36.78480556	36°	47'	05.3"	N	127°	57'	42.1"	127.9616944	E	J.W Seo	11.vii.2021	ON042021
Lep-545	*Falcileptoneta simboggulensis*	Outside simboggul cave: Galgeum-ri, Yeonpung-myeon, Goesan-gun, Chungcheongbuk-do, Korea	36.78480556	36°	47'	05.3"	N	127°	57'	42.1"	127.9616944	E	J.W Seo	11.vii.2021	ON042022
Lep-546	*Leptoneta taeguensis*	Mt. Daedeoksan: San62, Songhyeon-dong, Dalseo-gu, Daegu, Korea	35.82552778	35°	49'	31.9"	N	128°	33'	41.6"	128.5615556	E	J.H Oh	23.vii.2021	ON042023
Lep-547	*Leptoneta taeguensis*	Mt. Daedeoksan: San62, Songhyeon-dong, Dalseo-gu, Daegu, Korea	35.82552778	35°	49'	31.9"	N	128°	33'	41.6"	128.5615556	E	J.H Oh	23.vii.2021	ON042024
Lep-548	*Leptoneta taeguensis*	Mt. Daedeoksan: San62, Songhyeon-dong, Dalseo-gu, Daegu, Korea	35.82552778	35°	49'	31.9"	N	128°	33'	41.6"	128.5615556	E	J.H Oh	23.vii.2021	ON042025
Lep-549	*Leptoneta taeguensis*	Mt. Daedeoksan: San62, Songhyeon-dong, Dalseo-gu, Daegu, Korea	35.82552778	35°	49'	31.9"	N	128°	33'	41.6"	128.5615556	E	J.H Oh	23.vii.2021	ON042026
Lep-550	*Leptoneta taeguensis*	Mt. Daedeoksan: San62, Songhyeon-dong, Dalseo-gu, Daegu, Korea	35.82552778	35°	49'	31.9"	N	128°	33'	41.6"	128.5615556	E	J.H Oh	23.vii.2021	ON042027
Lep-551	*Leptoneta taeguensis*	Mt. Daedeoksan: San62, Songhyeon-dong, Dalseo-gu, Daegu, Korea	35.82552778	35°	49'	31.9"	N	128°	33'	41.6"	128.5615556	E	J.H Oh	23.vii.2021	ON042028
Lep-552	*Leptoneta taeguensis*	Mt. Daedeoksan: San62, Songhyeon-dong, Dalseo-gu, Daegu, Korea	35.82552778	35°	49'	31.9"	N	128°	33'	41.6"	128.5615556	E	J.H Oh	23.vii.2021	ON042029
Lep-553	*Leptoneta taeguensis*	825, Bangeum-ri, Unmun-myeon, Cheongdo-gun, Gyeongsangnam-do, Korea	35.69641667	35°	41'	47.1"	N	128°	56'	45.9"	128.9460833	E	J.H Oh	23.vii.2021	ON042030
Lep-554	*Leptoneta taeguensis*	825, Bangeum-ri, Unmun-myeon, Cheongdo-gun, Gyeongsangnam-do, Korea	35.69641667	35°	41'	47.1"	N	128°	56'	45.9"	128.9460833	E	J.H Oh	23.vii.2021	ON042031
Lep-555	*Leptoneta taeguensis*	825, Bangeum-ri, Unmun-myeon, Cheongdo-gun, Gyeongsangnam-do, Korea	35.69641667	35°	41'	47.1"	N	128°	56'	45.9"	128.9460833	E	J.H Oh	23.vii.2021	ON042032
Lep-556	*Leptoneta taeguensis*	825, Bangeum-ri, Unmun-myeon, Cheongdo-gun, Gyeongsangnam-do, Korea	35.69641667	35°	41'	47.1"	N	128°	56'	45.9"	128.9460833	E	J.H Oh	23.vii.2021	ON042033
Lep-557	*Leptoneta taeguensis*	825, Bangeum-ri, Unmun-myeon, Cheongdo-gun, Gyeongsangnam-do, Korea	35.69641667	35°	41'	47.1"	N	128°	56'	45.9"	128.9460833	E	J.H Oh	23.vii.2021	ON042034
Lep-558	*Leptoneta taeguensis*	Gucheon-ri, Danjang-myeon, Miryang-si, Gyeongsangnam-do, Korea	35.557	35°	33'	25.2"	N	128°	56'	21.0"	128.9391667	E	J.H Oh	23.vii.2021	ON042035
Lep-559	*Leptoneta taeguensis*	Gucheon-ri, Danjang-myeon, Miryang-si, Gyeongsangnam-do, Korea	35.557	35°	33'	25.2"	N	128°	56'	21.0"	128.9391667	E	J.H Oh	23.vii.2021	ON042036
Lep-560	*Leptoneta taeguensis*	Gucheon-ri, Danjang-myeon, Miryang-si, Gyeongsangnam-do, Korea	35.557	35°	33'	25.2"	N	128°	56'	21.0"	128.9391667	E	J.H Oh	23.vii.2021	ON042037
Lep-561	*Leptoneta taeguensis*	Gucheon-ri, Danjang-myeon, Miryang-si, Gyeongsangnam-do, Korea	35.557	35°	33'	25.2"	N	128°	56'	21.0"	128.9391667	E	J.H Oh	23.vii.2021	ON042038
Lep-562	*Leptoneta taeguensis*	Gucheon-ri, Danjang-myeon, Miryang-si, Gyeongsangnam-do, Korea	35.557	35°	33'	25.2"	N	128°	56'	21.0"	128.9391667	E	J.H Oh	23.vii.2021	ON042039
Lep-563	*Leptoneta taeguensis*	Gucheon-ri, Danjang-myeon, Miryang-si, Gyeongsangnam-do, Korea	35.557	35°	33'	25.2"	N	128°	56'	21.0"	128.9391667	E	J.H Oh	23.vii.2021	ON042040
Lep-564	*Leptoneta taeguensis*	Mt. Chunseongsan, Hogye-dong, Yangsan-si, Gyeongsangnam-do, Korea	35.38452778	35°	23'	04.3"	N	129°	06'	07.2"	129.102	E	J.H Oh	23.vii.2021	ON042041
Lep-565	*Leptoneta taeguensis*	Mt. Chunseongsan, Hogye-dong, Yangsan-si, Gyeongsangnam-do, Korea	35.38452778	35°	23'	04.3"	N	129°	06'	07.2"	129.102	E	J.H Oh	23.vii.2021	ON042042
Lep-566	*Leptoneta taeguensis*	Icheon-ri, Sangbuk-myeon, Ulju-gun, Ulsan, Korea	35.56188889	35°	33'	42.8"	N	129°	01'	31.0"	129.0252778	E	J.H Oh	24.vii.2021	ON042043
Lep-567	*Leptoneta taeguensis*	Icheon-ri, Sangbuk-myeon, Ulju-gun, Ulsan, Korea	35.56188889	35°	33'	42.8"	N	129°	01'	31.0"	129.0252778	E	J.H Oh	24.vii.2021	ON042044
Lep-568	*Leptoneta taeguensis*	Icheon-ri, Sangbuk-myeon, Ulju-gun, Ulsan, Korea	35.56188889	35°	33'	42.8"	N	129°	01'	31.0"	129.0252778	E	J.H Oh	24.vii.2021	ON042045
Lep-569	*Leptoneta taeguensis*	Icheon-ri, Sangbuk-myeon, Ulju-gun, Ulsan, Korea	35.56188889	35°	33'	42.8"	N	129°	01'	31.0"	129.0252778	E	J.H Oh	24.vii.2021	ON042046
Lep-570	*Leptoneta taeguensis*	Icheon-ri, Sangbuk-myeon, Ulju-gun, Ulsan, Korea	35.56188889	35°	33'	42.8"	N	129°	01'	31.0"	129.0252778	E	J.H Oh	24.vii.2021	ON042047
Lep-571	*Leptoneta taeguensis*	Icheon-ri, Sangbuk-myeon, Ulju-gun, Ulsan, Korea	35.56188889	35°	33'	42.8"	N	129°	01'	31.0"	129.0252778	E	J.H Oh	24.vii.2021	ON042048
Lep-572	*Falcileptoneta unmunensis*	Mt. Yeomposan, Bangeo-dong, Dong-gu, Ulsan, Korea	35.51825	35°	31'	05.7"	N	129°	24'	00.2"	129.4000556	E	J.H Oh	25.vii.2021	ON042049
Lep-573	*Falcileptoneta unmunensis*	Mt. Yeomposan, Bangeo-dong, Dong-gu, Ulsan, Korea	35.51825	35°	31'	05.7"	N	129°	24'	00.2"	129.4000556	E	J.H Oh	25.vii.2021	ON042050
Lep-574	*Falcileptoneta unmunensis*	Mt. Yeomposan, Bangeo-dong, Dong-gu, Ulsan, Korea	35.51825	35°	31'	05.7"	N	129°	24'	00.2"	129.4000556	E	J.H Oh	25.vii.2021	ON042051
Lep-576	*Leptoneta chilbosanensis*	Mt. Chilbosan: Cheoncheon-ri, Maesong-myeon, Hwaseong-si, Gyeonggi-do, Korea	37.25280556	37°	15'	10.1"	N	126°	56'	28.5"	126.94125	E	J.H Oh	27.vii.2021	ON042052
Lep-578	*Leptoneta chilbosanensis*	Mt. Chilbosan: Cheoncheon-ri, Maesong-myeon, Hwaseong-si, Gyeonggi-do, Korea	37.25280556	37°	15'	10.1"	N	126°	56'	28.5"	126.94125	E	J.H Oh	27.vii.2021	ON042053
Lep-579	*Leptoneta chilbosanensis*	Mt. Chilbosan: Cheoncheon-ri, Maesong-myeon, Hwaseong-si, Gyeonggi-do, Korea	37.25280556	37°	15'	10.1"	N	126°	56'	28.5"	126.94125	E	J.H Oh	27.vii.2021	ON042054
Lep-580	*Leptoneta chilbosanensis*	Mt. Chilbosan: Cheoncheon-ri, Maesong-myeon, Hwaseong-si, Gyeonggi-do, Korea	37.25280556	37°	15'	10.1"	N	126°	56'	28.5"	126.94125	E	J.H Oh	27.vii.2021	ON042055
Lep-581	*Leptoneta chilbosanensis*	Mt. Chilbosan: Cheoncheon-ri, Maesong-myeon, Hwaseong-si, Gyeonggi-do, Korea	37.25280556	37°	15'	10.1"	N	126°	56'	28.5"	126.94125	E	J.H Oh	27.vii.2021	ON042056
Lep-582	*Leptoneta chilbosanensis*	Mt. Chilbosan: Cheoncheon-ri, Maesong-myeon, Hwaseong-si, Gyeonggi-do, Korea	37.25280556	37°	15'	10.1"	N	126°	56'	28.5"	126.94125	E	J.H Oh	27.vii.2021	ON042057
Lep-583	*Leptoneta chilbosanensis*	Mt. Chilbosan: Cheoncheon-ri, Maesong-myeon, Hwaseong-si, Gyeonggi-do, Korea	37.25280556	37°	15'	10.1"	N	126°	56'	28.5"	126.94125	E	J.H Oh	27.vii.2021	ON042058
Lep-584	*Leptoneta chilbosanensis*	Mt. Chilbosan: Cheoncheon-ri, Maesong-myeon, Hwaseong-si, Gyeonggi-do, Korea	37.25280556	37°	15'	10.1"	N	126°	56'	28.5"	126.94125	E	J.H Oh	27.vii.2021	ON042059
Lep-585	*Leptoneta chilbosanensis*	Mt. Chilbosan: Cheoncheon-ri, Maesong-myeon, Hwaseong-si, Gyeonggi-do, Korea	37.25280556	37°	15'	10.1"	N	126°	56'	28.5"	126.94125	E	J.H Oh	27.vii.2021	ON042060
Lep-586	*Leptoneta chilbosanensis*	Mt. Chilbosan: Cheoncheon-ri, Maesong-myeon, Hwaseong-si, Gyeonggi-do, Korea	37.25280556	37°	15'	10.1"	N	126°	56'	28.5"	126.94125	E	J.H Oh	27.vii.2021	ON042061
Lep-587	*Leptoneta chilbosanensis*	Mt. Chilbosan: Cheoncheon-ri, Maesong-myeon, Hwaseong-si, Gyeonggi-do, Korea	37.25280556	37°	15'	10.1"	N	126°	56'	28.5"	126.94125	E	J.H Oh	27.vii.2021	ON042062
Lep-588	*Leptoneta chilbosanensis*	Mt. Chilbosan: Cheoncheon-ri, Maesong-myeon, Hwaseong-si, Gyeonggi-do, Korea	37.25280556	37°	15'	10.1"	N	126°	56'	28.5"	126.94125	E	J.H Oh	27.vii.2021	ON042063
Lep-589	*Leptoneta chilbosanensis*	Mt. Chilbosan: Cheoncheon-ri, Maesong-myeon, Hwaseong-si, Gyeonggi-do, Korea	37.25280556	37°	15'	10.1"	N	126°	56'	28.5"	126.94125	E	J.H Oh	27.vii.2021	ON042064
Lep-590	*Leptoneta chilbosanensis*	Mt. Chilbosan: Cheoncheon-ri, Maesong-myeon, Hwaseong-si, Gyeonggi-do, Korea	37.25280556	37°	15'	10.1"	N	126°	56'	28.5"	126.94125	E	J.H Oh	27.vii.2021	ON042065
Lep-597	*Leptoneta namhensis*	Danghang-ri, Nam-myeon, Namhae-gun, Gyeongsangnam-do, Korea	34.77055556	34°	46'	14.0"	N	127°	54'	39.0"	127.9108333	E	J.H Oh	02.viii.2021	ON042066
Lep-600	*Leptoneta paikmyeonggulensis*	Sinjeon-ri, Idong-myeon, Namhae-gun, Gyeongsangnam-do, Korea	34.77747222	34°	46'	38.9"	N	127°	58'	00.6"	127.9668333	E	J.H Oh	03.viii.2021	ON042067
Lep-601	*Leptoneta paikmyeonggulensis*	Sinjeon-ri, Idong-myeon, Namhae-gun, Gyeongsangnam-do, Korea	34.77747222	34°	46'	38.9"	N	127°	58'	00.6"	127.9668333	E	J.H Oh	03.viii.2021	ON042068
Lep-602	*Falcileptoneta geumsanensis*	Sinjeon-ri, Idong-myeon, Namhae-gun, Gyeongsangnam-do, Korea	34.74013889	34°	44'	24.5"	N	127°	59'	00.0"	127.9833333	E	J.H Oh	03.viii.2021	ON042069
Lep-611	*Falcileptoneta geumsanensis*	Sinjeon-ri, Idong-myeon, Namhae-gun, Gyeongsangnam-do, Korea	34.74013889	34°	44'	24.5"	N	127°	59'	00.0"	127.9833333	E	J.H Oh	03.viii.2021	ON042070
Lep-612	*Falcileptoneta geumsanensis*	Sinjeon-ri, Idong-myeon, Namhae-gun, Gyeongsangnam-do, Korea	34.74013889	34°	44'	24.5"	N	127°	59'	00.0"	127.9833333	E	J.H Oh	03.viii.2021	ON042071
Lep-614	*Leptoneta paikmyeonggulensis*	Sinjeon-ri, Idong-myeon, Namhae-gun, Gyeongsangnam-do, Korea	34.74013889	34°	44'	24.5"	N	127°	59'	00.0"	127.9833333	E	J.H Oh	03.viii.2021	ON042072

Lep-615	*Falcileptoneta geumsanensis*	Sinjeon-ri, Idong-myeon, Namhae-gun, Gyeongsangnam-do, Korea	34.74013889	34°	44’	24.5”	N	127°	59’	00.0”	127.9833333	E	J.H Oh	03.viii.2021	ON042073
Lep-617	*Falcileptoneta geumsanensis*	Sinjeon-ri, Idong-myeon, Namhae-gun, Gyeongsangnam-do, Korea	34.74013889	34°	44’	24.5”	N	127°	59’	00.0”	127.9833333	E	J.H Oh	03.viii.2021	ON042074
Lep-618	*Falcileptoneta geumsanensis*	Sinjeon-ri, Idong-myeon, Namhae-gun, Gyeongsangnam-do, Korea	34.74013889	34°	44’	24.5”	N	127°	59’	00.0”	127.9833333	E	J.H Oh	03.viii.2021	ON042075
Lep-619	*Falcileptoneta geumsanensis*	Sinjeon-ri, Idong-myeon, Namhae-gun, Gyeongsangnam-do, Korea	34.74013889	34°	44’	24.5”	N	127°	59’	00.0”	127.9833333	E	J.H Oh	03.viii.2021	ON042076
Lep-620	*Falcileptoneta geumsanensis*	Sinjeon-ri, Idong-myeon, Namhae-gun, Gyeongsangnam-do, Korea	34.74013889	34°	44’	24.5”	N	127°	59’	00.0”	127.9833333	E	J.H Oh	03.viii.2021	ON042077
Lep-621	*Falcileptoneta geumsanensis*	Sinjeon-ri, Idong-myeon, Namhae-gun, Gyeongsangnam-do, Korea	34.74013889	34°	44’	24.5”	N	127°	59’	00.0”	127.9833333	E	J.H Oh	03.viii.2021	ON042078
Lep-622	*Falcileptoneta geumsanensis*	Sinjeon-ri, Idong-myeon, Namhae-gun, Gyeongsangnam-do, Korea	34.74013889	34°	44’	24.5”	N	127°	59’	00.0”	127.9833333	E	J.H Oh	03.viii.2021	ON042079
Lep-623	*Falcileptoneta* sp23	Yeong-un-ri, Sanyang-eup, Tongyeong-si, Gyeongsangnam-do, Korea	34.80972222	34°	48’	35.0”	N	128°	25’	01.0”	128.4169444	E	J.H Oh	04.viii.2021	ON042080
Lep-624	*Falcileptoneta* sp23	Yeong-un-ri, Sanyang-eup, Tongyeong-si, Gyeongsangnam-do, Korea	34.80972222	34°	48’	35.0”	N	128°	25’	01.0”	128.4169444	E	J.H Oh	04.viii.2021	ON042081
Lep-625	*Falcileptoneta hansanensis*	Yeong-un-ri, Sanyang-eup, Tongyeong-si, Gyeongsangnam-do, Korea	34.80972222	34°	48’	35.0”	N	128°	25’	01.0”	128.4169444	E	J.H Oh	04.viii.2021	ON042082
Lep-626	*Falcileptoneta hansanensis*	Yeong-un-ri, Sanyang-eup, Tongyeong-si, Gyeongsangnam-do, Korea	34.80972222	34°	48’	35.0”	N	128°	25’	01.0”	128.4169444	E	J.H Oh	04.viii.2021	ON042083
Lep-627	*Falcileptoneta hansanensis*	Yeong-un-ri, Sanyang-eup, Tongyeong-si, Gyeongsangnam-do, Korea	34.80972222	34°	48’	35.0”	N	128°	25’	01.0”	128.4169444	E	J.H Oh	04.viii.2021	ON042084
Lep-628	*Falcileptoneta hansanensis*	Yeong-un-ri, Sanyang-eup, Tongyeong-si, Gyeongsangnam-do, Korea	34.80972222	34°	48’	35.0”	N	128°	25’	01.0”	128.4169444	E	J.H Oh	04.viii.2021	ON042085
Lep-629	*Falcileptoneta hansanensis*	Yeong-un-ri, Sanyang-eup, Tongyeong-si, Gyeongsangnam-do, Korea	34.80972222	34°	48’	35.0”	N	128°	25’	01.0”	128.4169444	E	J.H Oh	04.viii.2021	ON042086
Lep-632	*Leptoneta kwangreungensis*	Bupyeong-ri, Jinjeon-eup, Namyangju-si, Gyeonggi-do, Korea	37.74944444	37°	44’	58.0”	N	127°	10’	17.0”	127.1713889	E	J.H Oh	09.vii.2021	ON042087
Lep-633	*Leptoneta kwangreungensis*	Bupyeong-ri, Jinjeon-eup, Namyangju-si, Gyeonggi-do, Korea	37.74944444	37°	44’	58.0”	N	127°	10’	17.0”	127.1713889	E	J.H Oh	09.vii.2021	ON042088
Lep-634	*Leptoneta kwangreungensis*	Bupyeong-ri, Jinjeon-eup, Namyangju-si, Gyeonggi-do, Korea	37.74944444	37°	44’	58.0”	N	127°	10’	17.0”	127.1713889	E	J.H Oh	09.vii.2021	ON042089
Lep-636	*Leptoneta spinipalpus*	Bupyeong-ri, Jinjeon-eup, Namyangju-si, Gyeonggi-do, Korea	37.74944444	37°	44’	58.0”	N	127°	10’	17.0”	127.1713889	E	J.H Oh	09.vii.2021	ON042090
Lep-637	*Leptoneta spinipalpus*	Bupyeong-ri, Jinjeon-eup, Namyangju-si, Gyeonggi-do, Korea	37.74944444	37°	44’	58.0”	N	127°	10’	17.0”	127.1713889	E	J.H Oh	09.vii.2021	ON042091
Lep-638	*Falcileptoneta* sp19	Hakgye-ri, Haksan-myeon, Yeongam-gun, Jeollanam-do, Korea	34.69494444	34°	41’	41.8”	N	126°	37’	53.2”	126.6314444	E	J.H Oh	08.ix.2021	ON042092
Lep-639	*Falcileptoneta* sp19	Hakgye-ri, Haksan-myeon, Yeongam-gun, Jeollanam-do, Korea	34.69494444	34°	41’	41.8”	N	126°	37’	53.2”	126.6314444	E	J.H Oh	08.ix.2021	ON042093
Lep-640	*Falcileptoneta* sp9	Sacheon-ri, Uisin-myeon, Jindo-gun, Jeollanam-do, Korea	34.46605556	34°	27’	57.8”	N	126°	18’	19.2”	126.3053333	E	J.H Oh	08.ix.2021	ON042094
Lep-641	*Falcileptoneta* sp9	Sacheon-ri, Uisin-myeon, Jindo-gun, Jeollanam-do, Korea	34.46605556	34°	27’	57.8”	N	126°	18’	19.2”	126.3053333	E	J.H Oh	08.ix.2021	ON042095
Lep-642	*Longileptoneta* sp9	Sacheon-ri, Uisin-myeon, Jindo-gun, Jeollanam-do, Korea	34.46605556	34°	27’	57.8”	N	126°	18’	19.2”	126.3053333	E	J.H Oh	08.ix.2021	ON042096
Lep-643	*Longileptoneta* sp9	Sacheon-ri, Uisin-myeon, Jindo-gun, Jeollanam-do, Korea	34.46605556	34°	27’	57.8”	N	126°	18’	19.2”	126.3053333	E	J.H Oh	08.ix.2021	ON042097
Lep-644	*Falcileptoneta* sp17	Mt. Duryunsan: Gurim-ri, Samsan-myeon, Haenam-gun, Jeollanam-do, Korea	34.483	34°	28’	58.8”	N	126°	36’	51.6”	126.6143333	E	J.H Oh	09.ix.2021	ON042098
Lep-645	*Falcileptoneta* sp17	Mt. Duryunsan: Gurim-ri, Samsan-myeon, Haenam-gun, Jeollanam-do, Korea	34.483	34°	28’	58.8”	N	126°	36’	51.6”	126.6143333	E	J.H Oh	09.ix.2021	ON042099
Lep-646	*Falcileptoneta* sp17	Mt. Duryunsan: Gurim-ri, Samsan-myeon, Haenam-gun, Jeollanam-do, Korea	34.483	34°	28’	58.8”	N	126°	36’	51.6”	126.6143333	E	J.H Oh	09.ix.2021	ON042100
Lep-648	*Falcileptoneta* sp17	Mt. Duryunsan: Gurim-ri, Samsan-myeon, Haenam-gun, Jeollanam-do, Korea	34.483	34°	28’	58.8”	N	126°	36’	51.6”	126.6143333	E	J.H Oh	09.ix.2021	ON042101
Lep-650	*Falcileptoneta* sp17	Mt. Duryunsan: Gurim-ri, Samsan-myeon, Haenam-gun, Jeollanam-do, Korea	34.483	34°	28’	58.8”	N	126°	36’	51.6”	126.6143333	E	J.H Oh	09.ix.2021	ON042102
Lep-652	*Falcileptoneta* sp8	Wando Arboretum: 156, Chopyeong 1-gil, Gunoe-myeon, Wando-gun, Jeollanam-do, Korea	34.36016667	34°	21’	36.6”	N	126°	39’	41.8”	126.6616111	E	J.H Oh	09.ix.2021	ON042103
Lep-661	*Falcileptoneta* sp8	Wando Arboretum: 156, Chopyeong 1-gil, Gunoe-myeon, Wando-gun, Jeollanam-do, Korea	34.36016667	34°	21’	36.6”	N	126°	39’	41.8”	126.6616111	E	J.H Oh	09.ix.2021	ON042104
Lep-662	*Falcileptoneta* sp8	Wando Arboretum: 156, Chopyeong 1-gil, Gunoe-myeon, Wando-gun, Jeollanam-do, Korea	34.36016667	34°	21’	36.6”	N	126°	39’	41.8”	126.6616111	E	J.H Oh	09.ix.2021	ON042105
Lep-664	*Falcileptoneta* sp8	Wando Arboretum: 156, Chopyeong 1-gil, Gunoe-myeon, Wando-gun, Jeollanam-do, Korea	34.36016667	34°	21’	36.6”	N	126°	39’	41.8”	126.6616111	E	J.H Oh	09.ix.2021	ON042106
Lep-665	*Falcileptoneta* sp8	Wando Arboretum: 156, Chopyeong 1-gil, Gunoe-myeon, Wando-gun, Jeollanam-do, Korea	34.36016667	34°	21’	36.6”	N	126°	39’	41.8”	126.6616111	E	J.H Oh	09.ix.2021	ON042107
Lep-666	*Falcileptoneta* sp8	Wando Arboretum: 156, Chopyeong 1-gil, Gunoe-myeon, Wando-gun, Jeollanam-do, Korea	34.36016667	34°	21’	36.6”	N	126°	39’	41.8”	126.6616111	E	J.H Oh	09.ix.2021	ON042108
Lep-667	*Falcileptoneta* sp8	Wando Arboretum: 156, Chopyeong 1-gil, Gunoe-myeon, Wando-gun, Jeollanam-do, Korea	34.36016667	34°	21’	36.6”	N	126°	39’	41.8”	126.6616111	E	J.H Oh	09.ix.2021	ON042109
Lep-668	*Falcileptoneta* sp8	Wando Arboretum: 156, Chopyeong 1-gil, Gunoe-myeon, Wando-gun, Jeollanam-do, Korea	34.36016667	34°	21’	36.6”	N	126°	39’	41.8”	126.6616111	E	J.H Oh	09.ix.2021	ON042110
Lep-669	*Falcileptoneta* sp8	Wando Arboretum: 156, Chopyeong 1-gil, Gunoe-myeon, Wando-gun, Jeollanam-do, Korea	34.36016667	34°	21’	36.6”	N	126°	39’	41.8”	126.6616111	E	J.H Oh	09.ix.2021	ON042111
Lep-670	*Falcileptoneta* sp17	Wando Arboretum: 156, Chopyeong 1-gil, Gunoe-myeon, Wando-gun, Jeollanam-do, Korea	34.36016667	34°	21’	36.6”	N	126°	39’	41.8”	126.6616111	E	J.H Oh	09.ix.2021	ON042112
Lep-671	*Falcileptoneta* sp17	Wando Arboretum: 156, Chopyeong 1-gil, Gunoe-myeon, Wando-gun, Jeollanam-do, Korea	34.36016667	34°	21’	36.6”	N	126°	39’	41.8”	126.6616111	E	J.H Oh	09.ix.2021	ON042113
Lep-673	*Falcileptoneta* sp17	Wando Arboretum: 156, Chopyeong 1-gil, Gunoe-myeon, Wando-gun, Jeollanam-do, Korea	34.36016667	34°	21’	36.6”	N	126°	39’	41.8”	126.6616111	E	J.H Oh	09.ix.2021	ON042114
Lep-674	*Falcileptoneta* sp17	Wando Arboretum: 156, Chopyeong 1-gil, Gunoe-myeon, Wando-gun, Jeollanam-do, Korea	34.36016667	34°	21’	36.6”	N	126°	39’	41.8”	126.6616111	E	J.H Oh	09.ix.2021	ON042115
Lep-675	*Falcileptoneta* sp17	Wando Arboretum: 156, Chopyeong 1-gil, Gunoe-myeon, Wando-gun, Jeollanam-do, Korea	34.36016667	34°	21’	36.6”	N	126°	39’	41.8”	126.6616111	E	J.H Oh	09.ix.2021	ON042116
Lep-678	*Falcileptoneta* sp17	Wando Arboretum: 156, Chopyeong 1-gil, Gunoe-myeon, Wando-gun, Jeollanam-do, Korea	34.36016667	34°	21’	36.6”	N	126°	39’	41.8”	126.6616111	E	J.H Oh	09.ix.2021	ON042117
Lep-679	*Falcileptoneta* sp17	Wando Arboretum: 156, Chopyeong 1-gil, Gunoe-myeon, Wando-gun, Jeollanam-do, Korea	34.36016667	34°	21’	36.6”	N	126°	39’	41.8”	126.6616111	E	J.H Oh	09.ix.2021	ON042118
Lep-680	*Falcileptoneta* sp17	Wando Arboretum: 156, Chopyeong 1-gil, Gunoe-myeon, Wando-gun, Jeollanam-do, Korea	34.36016667	34°	21’	36.6”	N	126°	39’	41.8”	126.6616111	E	J.H Oh	09.ix.2021	ON042119
Lep-681	*Falcileptoneta* sp17	Wando Arboretum: 156, Chopyeong 1-gil, Gunoe-myeon, Wando-gun, Jeollanam-do, Korea	34.36016667	34°	21’	36.6”	N	126°	39’	41.8”	126.6616111	E	J.H Oh	09.ix.2021	ON042120
Lep-682	*Falcileptoneta* sp17	Wando Arboretum: 156, Chopyeong 1-gil, Gunoe-myeon, Wando-gun, Jeollanam-do, Korea	34.36016667	34°	21’	36.6”	N	126°	39’	41.8”	126.6616111	E	J.H Oh	09.ix.2021	ON042121
Lep-683	*Falcileptoneta* sp17	Wando Arboretum: 156, Chopyeong 1-gil, Gunoe-myeon, Wando-gun, Jeollanam-do, Korea	34.36016667	34°	21’	36.6”	N	126°	39’	41.8”	126.6616111	E	J.H Oh	09.ix.2021	ON042122
Lep-685	*Falcileptoneta* sp16	Paryeong-ro, Yeongnam-myeon, Goheung-gun, Jeollanam-do, Korea	34.62316667	34°	37’	23.4”	N	127°	27’	05.8”	127.4516111	E	J.H Oh	10.ix.2021	ON042123
Lep-686	*Falcileptoneta* sp16	Paryeong-ro, Yeongnam-myeon, Goheung-gun, Jeollanam-do, Korea	34.62316667	34°	37’	23.4”	N	127°	27’	05.8”	127.4516111	E	J.H Oh	10.ix.2021	ON042124
Lep-687	*Falcileptoneta* sp16	Paryeong-ro, Yeongnam-myeon, Goheung-gun, Jeollanam-do, Korea	34.62316667	34°	37’	23.4”	N	127°	27’	05.8”	127.4516111	E	J.H Oh	10.ix.2021	ON042125
Lep-688	*Leptoneta* sp1	Paryeong-ro, Yeongnam-myeon, Goheung-gun, Jeollanam-do, Korea	34.62316667	34°	37’	23.4”	N	127°	27’	05.8”	127.4516111	E	J.H Oh	10.ix.2021	ON042126
Lep-689	*Falcileptoneta* sp16	Paryeong-ro, Yeongnam-myeon, Goheung-gun, Jeollanam-do, Korea	34.62316667	34°	37’	23.4”	N	127°	27’	05.8”	127.4516111	E	J.H Oh	10.ix.2021	ON042127
Lep-690	*Falcileptoneta* sp16	Paryeong-ro, Yeongnam-myeon, Goheung-gun, Jeollanam-do, Korea	34.62316667	34°	37’	23.4”	N	127°	27’	05.8”	127.4516111	E	J.H Oh	10.ix.2021	ON042128
Lep-691	*Leptoneta* sp1	Paryeong-ro, Yeongnam-myeon, Goheung-gun, Jeollanam-do, Korea	34.62316667	34°	37’	23.4”	N	127°	27’	05.8”	127.4516111	E	J.H Oh	10.ix.2021	ON042129
Lep-692	*Falcileptoneta* sp17	Geogeumdo Island: 293, Singpyeong-ri, Geumsan-myeon, Goheung-gun, Jeollanam-do, Korea	34.45311111	34°	27’	11.2”	N	127°	13’	04.8”	127.218	E	J.H Oh	10.ix.2021	ON042130
Lep-693	*Falcileptoneta* sp17	Geogeumdo Island: 293, Singpyeong-ri, Geumsan-myeon, Goheung-gun, Jeollanam-do, Korea	34.45311111	34°	27’	11.2”	N	127°	13’	04.8”	127.218	E	J.H Oh	10.ix.2021	ON042131
Lep-695	*Falcileptoneta* sp17	Geogeumdo Island: 293, Singpyeong-ri, Geumsan-myeon, Goheung-gun, Jeollanam-do, Korea	34.45311111	34°	27’	11.2”	N	127°	13’	04.8”	127.218	E	J.H Oh	10.ix.2021	ON042132
Lep-696	*Falcileptoneta* sp17	Geogeumdo Island: 293, Singpyeong-ri, Geumsan-myeon, Goheung-gun, Jeollanam-do, Korea	34.45311111	34°	27’	11.2”	N	127°	13’	04.8”	127.218	E	J.H Oh	10.ix.2021	ON042133
Lep-697	*Falcileptoneta* sp17	Geogeumdo Island: 293, Singpyeong-ri, Geumsan-myeon, Goheung-gun, Jeollanam-do, Korea	34.45311111	34°	27’	11.2”	N	127°	13’	04.8”	127.218	E	J.H Oh	10.ix.2021	ON042134
Lep-700	*Falcileptoneta naejangensis*	Mt. Jogyesan: Seokheung-ri, Nagan-myeon, Suncheon-si, Jeollanam-do, Korea	34.95222222	34°	57’	08.0”	N	127°	20’	45.0”	127.3458333	E	J.H Oh	11.ix.2021	ON042135
Lep-702	*Longileptoneta byeonsanbando*	Udong-ri, Boan-myeon, Buan-gun, Jeollabuk-do, Korea	35.62811111	35°	37’	41.2”	N	126°	37’	57.8”	126.6327222	E	J.H Oh	11.ix.2021	ON042136
Lep-703	*Longileptoneta byeonsanbando*	Udong-ri, Boan-myeon, Buan-gun, Jeollabuk-do, Korea	35.62811111	35°	37’	41.2”	N	126°	37’	57.8”	126.6327222	E	J.H Oh	11.ix.2021	ON042137
Lep-704	*Longileptoneta byeonsanbando*	Udong-ri, Boan-myeon, Buan-gun, Jeollabuk-do, Korea	35.62811111	35°	37’	41.2”	N	126°	37’	57.8”	126.6327222	E	J.H Oh	11.ix.2021	ON042138
Lep-705	*Longileptoneta byeonsanbando*	Udong-ri, Boan-myeon, Buan-gun, Jeollabuk-do, Korea	35.62811111	35°	37’	41.2”	N	126°	37’	57.8”	126.6327222	E	J.H Oh	11.ix.2021	ON042139
Lep-706	*Longileptoneta byeonsanbando*	Udong-ri, Boan-myeon, Buan-gun, Jeollabuk-do, Korea	35.62811111	35°	37’	41.2”	N	126°	37’	57.8”	126.6327222	E	J.H Oh	11.ix.2021	ON042140
Lep-707	*Longileptoneta byeonsanbando*	Udong-ri, Boan-myeon, Buan-gun, Jeollabuk-do, Korea	35.62811111	35°	37’	41.2”	N	126°	37’	57.8”	126.6327222	E	J.H Oh	11.ix.2021	ON042141
Lep-708	*Longileptoneta byeonsanbando*	Udong-ri, Boan-myeon, Buan-gun, Jeollabuk-do, Korea	35.62811111	35°	37’	41.2”	N	126°	37’	57.8”	126.6327222	E	J.H Oh	11.ix.2021	ON042142
Lep-709	*Longileptoneta byeonsanbando*	Udong-ri, Boan-myeon, Buan-gun, Jeollabuk-do, Korea	35.62811111	35°	37’	41.2”	N	126°	37’	57.8”	126.6327222	E	J.H Oh	11.ix.2021	ON042143
Lep-710	*Longileptoneta byeonsanbando*	Udong-ri, Boan-myeon, Buan-gun, Jeollabuk-do, Korea	35.62811111	35°	37’	41.2”	N	126°	37’	57.8”	126.6327222	E	J.H Oh	11.ix.2021	ON042144
Lep-718	*Longileptoneta* sp4	Daejangdo-Island: Daejangdo-ri, Okdo-myeon, Gunsan-si, Jeollabuk-do, Korea	35.81672222	35°	49’	00.2”	N	126°	23’	42.4”	126.3951111	E	J.H Oh	12.ix.2021	ON042145
Lep-719	*Longileptoneta* sp4	Daejangdo-Island: Daejangdo-ri, Okdo-myeon, Gunsan-si, Jeollabuk-do, Korea	35.81672222	35°	49’	00.2”	N	126°	23’	42.4”	126.3951111	E	J.H Oh	12.ix.2021	ON042146
Lep-720	*Falcileptoneta odaesanensis*	Mt. Odaesan: Samsan-ri, Yeongok-myeon, Gangneung-si, Gangwon-do, Korea	37.82677778	37°	49’	36.4”	N	128°	37’	51.8”	128.6310556	E	J.H Oh	15.ix.2021	ON042147
Lep-721	*Falcileptoneta odaesanensis*	Mt. Odaesan: Samsan-ri, Yeongok-myeon, Gangneung-si, Gangwon-do, Korea	37.82677778	37°	49’	36.4”	N	128°	37’	51.8”	128.6310556	E	J.H Oh	15.ix.2021	ON042148
Lep-722	*Falcileptoneta odaesanensis*	Mt. Odaesan: Myeonggae-ri, Nae-myeon, Hongcheon-gun, Gangwon-do, Korea	37.84052778	37°	50’	25.9”	N	128°	33’	03.5”	128.5509722	E	J.H Oh	16.ix.2021	ON042149
Lep-723	*Falcileptoneta odaesanensis*	Mt. Odaesan: Myeonggae-ri, Nae-myeon, Hongcheon-gun, Gangwon-do, Korea	37.84052778	37°	50’	25.9”	N	128°	33’	03.5”	128.5509722	E	J.H Oh	16.ix.2021	ON042150
Lep-724	*Falcileptoneta odaesanensis*	Mt. Odaesan: Myeonggae-ri, Nae-myeon, Hongcheon-gun, Gangwon-do, Korea	37.84052778	37°	50’	25.9”	N	128°	33’	03.5”	128.5509722	E	J.H Oh	16.ix.2021	ON042151
Lep-725	*Falcileptoneta odaesanensis*	Mt. Odaesan: Myeonggae-ri, Nae-myeon, Hongcheon-gun, Gangwon-do, Korea	37.84052778	37°	50’	25.9”	N	128°	33’	03.5”	128.5509722	E	J.H Oh	16.ix.2021	ON042152

Lep-726	*Falcileptoneta odaesanensis*	Mt. Odaesan: Myeonggae-ri, Nae-myeon, Hongcheon-gun, Gangwon-do, Korea	37.84052778	37°	50'	25.9"	N	128°	33'	03.5"	128.5509722	E	J.H Oh	16.ix.2021	ON042153
Lep-727	*Falcileptoneta odaesanensis*	Mt. Odaesan: Myeonggae-ri, Nae-myeon, Hongcheon-gun, Gangwon-do, Korea	37.84052778	37°	50'	25.9"	N	128°	33'	03.5"	128.5509722	E	J.H Oh	16.ix.2021	ON042154
Lep-728	*Leptoneta seogwipoensis*	Seonheul-ri, Jocheon-eup, Jeju-si, Jeju, Korea	33.47766667	33°	28'	39.6"	N	126°	42'	48.8"	126.7135556	E	S.W Yoon	18.vii.2021	ON042155
Lep-729	*Leptoneta seogwipoensis*	Seonheul-ri, Jocheon-eup, Jeju-si, Jeju, Korea	33.47766667	33°	28'	39.6"	N	126°	42'	48.8"	126.7135556	E	S.W Yoon	18.vii.2021	ON042156
Lep-730	*Falcileptoneta odaesanensis*	Mt. Jukbyeoksan: Yachon-ri, Jugwang-myeon, Goseong-gun, Gangwon-do, Korea	38.30675	38°	18'	24.3"	N	128°	29'	14.1"	128.48725	E	S.W Yoon	11.ix.2021	ON042157
Lep-731	*Falcileptoneta odaesanensis*	Mt. Jukbyeoksan: Yachon-ri, Jugwang-myeon, Goseong-gun, Gangwon-do, Korea	38.30666667	38°	18'	24.0"	N	128°	29'	14.1"	128.48725	E	S.W Yoon	11.ix.2021	ON042158
Lep-732	*Longileptoneta* sp3	Mt. Gyeryongsan: 567-3, Gapsa-ro, Gyeryong-myeon, Gongju-si, Chungcheongnam-do, Korea	36.36975	36°	22'	11.1"	N	127°	10'	53.0"	127.1813889	E	S.W Yoon	20.iv.2021	ON042159
Lep-734	*Longileptoneta* sp3	Mt. Gyeryongsan: 567-3, Gapsa-ro, Gyeryong-myeon, Gongju-si, Chungcheongnam-do, Korea	36.36975	36°	22'	11.1"	N	127°	10'	53.0"	127.1813889	E	S.W Yoon	20.iv.2021	ON042160
Lep-736	*Leptoneta spinipalpus*	Mt. Yumyeongsan: Gail-ri, Seorak-myeon, Gapyeong-gun, Gyeonggi-do, Korea	37.58888889	37°	35'	20.0"	N	127°	29'	18.1"	127.4883611	E	J.H Oh	24.ix.2021	ON042161
Lep-737	*Leptoneta spinipalpus*	Mt. Yumyeongsan: Gail-ri, Seorak-myeon, Gapyeong-gun, Gyeonggi-do, Korea	37.58888889	37°	35'	20.0"	N	127°	29'	18.1"	127.4883611	E	J.H Oh	24.ix.2021	ON042162
Lep-739	*Leptoneta spinipalpus*	Mt. Yumyeongsan: Gail-ri, Seorak-myeon, Gapyeong-gun, Gyeonggi-do, Korea	37.58888889	37°	35'	20.0"	N	127°	29'	18.1"	127.4883611	E	J.H Oh	24.ix.2021	ON042163
Lep-740	*Falcileptoneta geumdaensis*	Geumdae-ri, Panbu-myeon, Wonju-si, Gangwon-do, Korea	37.28	37°	16'	48.0"	N	128°	00'	55.0"	128.0152778	E	J.H Oh	30.ix.2021	ON042164
Lep-741	*Falcileptoneta odaesanensis*	Mt. Odaesan: Dongsan-ri, Jinbu-myeon, Pyeongchang-gun, Gangwon-do, Korea	37.78469444	37°	47'	04.9"	N	128°	34'	24.2"	128.5733889	E	J.H Oh	13.x.2021	ON042165
Lep-742	*Falcileptoneta odaesanensis*	Mt. Odaesan: Dongsan-ri, Jinbu-myeon, Pyeongchang-gun, Gangwon-do, Korea	37.78469444	37°	47'	04.9"	N	128°	34'	24.2"	128.5733889	E	J.H Oh	13.x.2021	ON042166
Lep-743	*Falcileptoneta odaesanensis*	Mt. Odaesan: Dongsan-ri, Jinbu-myeon, Pyeongchang-gun, Gangwon-do, Korea	37.78469444	37°	47'	04.9"	N	128°	34'	24.2"	128.5733889	E	J.H Oh	13.x.2021	ON042167
Lep-744	*Falcileptoneta odaesanensis*	Mt. Odaesan: Dongsan-ri, Jinbu-myeon, Pyeongchang-gun, Gangwon-do, Korea	37.78469444	37°	47'	04.9"	N	128°	34'	24.2"	128.5733889	E	J.H Oh	13.x.2021	ON042168
Lep-745	*Falcileptoneta chiakensis*	Mt. Gariwangsan: 115-2, Jangjeon-ri, Jinbu-myeon, Pyeongchang-gun, Gangwon-do, Korea	37.49166667	37°	29'	30.0"	N	128°	32'	31.0"	128.5419444	E	J.H Oh	14.x.2021	ON042169
Lep-747	*Leptoneta kwangreungensis*	Bupyeong-ri, Jinjeon-eup, Namyangju-si, Gyeonggi-do, Korea	37.74944444	37°	44'	58.0"	N	127°	10'	17.0"	127.1713889	E	J.H Oh	01.xi.2021	ON042170
Lep-749	*Falcileptoneta chiakensis*	Geumgul cave: Dodam-ri, Danyang-eup, Danyang-gun, Chungcheongbuk-do, Korea	36.99658333	36°	59'	47.7"	N	128°	21'	24.1"	128.3566944	E	J.G Lee & J.H Lee	25.ix.2021	ON042171
Lep-750	*Falcileptoneta chiakensis*	Geumgul cave: Dodam-ri, Danyang-eup, Danyang-gun, Chungcheongbuk-do, Korea	36.99658333	36°	59'	47.7"	N	128°	21'	24.1"	128.3566944	E	J.G Lee & J.H Lee	25.ix.2021	ON042172
Lep-751	*Longileptoneta* sp2	Ujeok Mine: 869-1 Sacheon-ri, Mongtan-myeon, Muan-gun, Jeollabuk-do, Korea	34.92916667	34°	55'	45.0"	N	126°	28'	08.0"	126.4688889	E	J.H Oh	12.xii.2021	ON042173
Lep-752	*Longileptoneta* sp2	Ujeok Mine: 869-1 Sacheon-ri, Mongtan-myeon, Muan-gun, Jeollabuk-do, Korea	34.92916667	34°	55'	45.0"	N	126°	28'	08.0"	126.4688889	E	J.H Oh	12.xii.2021	ON042174
Lep-753	*Longileptoneta* sp2	Ujeok Mine: 869-1 Sacheon-ri, Mongtan-myeon, Muan-gun, Jeollabuk-do, Korea	34.92916667	34°	55'	45.0"	N	126°	28'	08.0"	126.4688889	E	J.H Oh	12.xii.2021	ON042175
Lep-754	*Longileptoneta* sp2	Ujeok Mine: 869-1 Sacheon-ri, Mongtan-myeon, Muan-gun, Jeollabuk-do, Korea	34.92916667	34°	55'	45.0"	N	126°	28'	08.0"	126.4688889	E	J.H Oh	12.xii.2021	ON042176
Lep-755	*Longileptoneta* sp2	Ujeok Mine: 869-1 Sacheon-ri, Mongtan-myeon, Muan-gun, Jeollabuk-do, Korea	34.92916667	34°	55'	45.0"	N	126°	28'	08.0"	126.4688889	E	J.H Oh	12.xii.2021	ON042177
Lep-756	*Longileptoneta* sp2	Ujeok Mine: 869-1 Sacheon-ri, Mongtan-myeon, Muan-gun, Jeollabuk-do, Korea	34.92916667	34°	55'	45.0"	N	126°	28'	08.0"	126.4688889	E	J.H Oh	12.xii.2021	ON042178
Lep-757	*Longileptoneta* sp2	Ujeok Mine: 869-1 Sacheon-ri, Mongtan-myeon, Muan-gun, Jeollabuk-do, Korea	34.92916667	34°	55'	45.0"	N	126°	28'	08.0"	126.4688889	E	J.H Oh	12.xii.2021	ON042179
Lep-758	*Longileptoneta* sp2	Ujeok Mine: 869-1 Sacheon-ri, Mongtan-myeon, Muan-gun, Jeollabuk-do, Korea	34.92916667	34°	55'	45.0"	N	126°	28'	08.0"	126.4688889	E	J.H Oh	12.xii.2021	ON042180
Lep-759	*Longileptoneta* sp2	Ujeok Mine: 869-1 Sacheon-ri, Mongtan-myeon, Muan-gun, Jeollabuk-do, Korea	34.92916667	34°	55'	45.0"	N	126°	28'	08.0"	126.4688889	E	J.H Oh	12.xii.2021	ON042181
Lep-760	*Longileptoneta* sp2	Ujeok Mine: 869-1 Sacheon-ri, Mongtan-myeon, Muan-gun, Jeollabuk-do, Korea	34.92916667	34°	55'	45.0"	N	126°	28'	08.0"	126.4688889	E	J.H Oh	12.xii.2021	ON042182
Lep-761	*Falcileptoneta coreana*	Mosangul cave: Seongjeo-ri, Gaeun-eup, Mungyeong-si, Gyeongsangbuk-do, Korea	36.63136111	36°	37'	52.9"	N	128°	02'	10.2"	128.0361667	E	S.T Kim	31.viii.2020	ON042183
Lep-762	*Falcileptoneta coreana*	Mosangul cave: Seongjeo-ri, Gaeun-eup, Mungyeong-si, Gyeongsangbuk-do, Korea	36.63136111	36°	37'	52.9"	N	128°	02'	10.2"	128.0361667	E	S.T Kim	31.viii.2020	ON042184
Lep-763	*Falcileptoneta coreana*	Mosangul cave: Seongjeo-ri, Gaeun-eup, Mungyeong-si, Gyeongsangbuk-do, Korea	36.63136111	36°	37'	52.9"	N	128°	02'	10.2"	128.0361667	E	S.T Kim	31.viii.2020	ON042185
Lep-764	*Falcileptoneta coreana*	Mosangul cave: Seongjeo-ri, Gaeun-eup, Mungyeong-si, Gyeongsangbuk-do, Korea	36.63136111	36°	37'	52.9"	N	128°	02'	10.2"	128.0361667	E	S.T Kim	31.viii.2020	ON042186
Lep-765	*Falcileptoneta coreana*	Mosangul cave: Seongjeo-ri, Gaeun-eup, Mungyeong-si, Gyeongsangbuk-do, Korea	36.63136111	36°	37'	52.9"	N	128°	02'	10.2"	128.0361667	E	S.T Kim	31.viii.2020	ON042187
Lep-766	*Falcileptoneta coreana*	Mosangul cave: Seongjeo-ri, Gaeun-eup, Mungyeong-si, Gyeongsangbuk-do, Korea	36.63136111	36°	37'	52.9"	N	128°	02'	10.2"	128.0361667	E	S.T Kim	31.viii.2020	ON042188
Lep-767	*Falcileptoneta coreana*	Mosangul cave: Seongjeo-ri, Gaeun-eup, Mungyeong-si, Gyeongsangbuk-do, Korea	36.63136111	36°	37'	52.9"	N	128°	02'	10.2"	128.0361667	E	S.T Kim	31.viii.2020	ON042189
Lep-768	*Falcileptoneta coreana*	Mosangul cave: Seongjeo-ri, Gaeun-eup, Mungyeong-si, Gyeongsangbuk-do, Korea	36.63136111	36°	37'	52.9"	N	128°	02'	10.2"	128.0361667	E	S.T Kim	31.viii.2020	ON042190
Lep-769	*Falcileptoneta coreana*	Mosangul cave: Seongjeo-ri, Gaeun-eup, Mungyeong-si, Gyeongsangbuk-do, Korea	36.63136111	36°	37'	52.9"	N	128°	02'	10.2"	128.0361667	E	S.T Kim	31.viii.2020	ON042191
Lep-770	*Falcileptoneta coreana*	Mosangul cave: Seongjeo-ri, Gaeun-eup, Mungyeong-si, Gyeongsangbuk-do, Korea	36.63136111	36°	37'	52.9"	N	128°	02'	10.2"	128.0361667	E	S.T Kim	31.viii.2020	ON042192
Lep-771	*Falcileptoneta coreana*	Mosangul cave: Seongjeo-ri, Gaeun-eup, Mungyeong-si, Gyeongsangbuk-do, Korea	36.63136111	36°	37'	52.9"	N	128°	02'	10.2"	128.0361667	E	S.T Kim	31.viii.2020	ON042193
Lep-796	*Leptoneta handeulgulensis*	Heyeopjaegul cave: Hyeopjae-ri, Hallim-eup, Jeju-si, Jeju-do, Korea	33.38961111	33°	23'	22.6"	N	126°	14'	21.1"	126.2391944	E	Y.G Choi	28.vi.2021	ON042194
Lep-797	*Leptoneta handeulgulensis*	Heyeopjaegul cave: Hyeopjae-ri, Hallim-eup, Jeju-si, Jeju-do, Korea	33.38961111	33°	23'	22.6"	N	126°	14'	21.1"	126.2391944	E	Y.G Choi	28.vi.2021	ON042195
Lep-798	*Leptoneta handeulgulensis*	Heyeopjaegul cave: Hyeopjae-ri, Hallim-eup, Jeju-si, Jeju-do, Korea	33.38961111	33°	23'	22.6"	N	126°	14'	21.1"	126.2391944	E	Y.G Choi	28.vi.2021	ON042196
Lep-802	*Leptoneta handeulgulensis*	Heyeopjaegul cave: Hyeopjae-ri, Hallim-eup, Jeju-si, Jeju-do, Korea	33.38961111	33°	23'	22.6"	N	126°	14'	21.1"	126.2391944	E	Y.G Choi	28.vi.2021	ON042197
Lep-804	*Leptoneta handeulgulensis*	Heyeopjaegul cave: Hyeopjae-ri, Hallim-eup, Jeju-si, Jeju-do, Korea	33.38961111	33°	23'	22.6"	N	126°	14'	21.1"	126.2391944	E	Y.G Choi	28.vi.2021	ON042198
Lep-808	*Leptoneta hogyegulensis*	Hogyegul cave: Byeoram-ri, Hogye-myeon, Mungyeong-si, Gyeongsangbuk-do, Korea	36.66186111	36°	39'	42.7"	N	128°	10'	25.9"	128.1738611	E	Y.G Choi	23.vi.2019	ON042199
Lep-809	*Leptoneta hogyegulensis*	Hogyegul cave: Byeoram-ri, Hogye-myeon, Mungyeong-si, Gyeongsangbuk-do, Korea	36.66186111	36°	39'	42.7"	N	128°	10'	25.9"	128.1738611	E	Y.G Choi	23.vi.2019	ON042200
Lep-810	*Leptoneta hogyegulensis*	Hogyegul cave: Byeoram-ri, Hogye-myeon, Mungyeong-si, Gyeongsangbuk-do, Korea	36.66186111	36°	39'	42.7"	N	128°	10'	25.9"	128.1738611	E	Y.G Choi	23.vi.2019	ON042201
Lep-811	*Leptoneta hogyegulensis*	Hogyegul cave: Byeoram-ri, Hogye-myeon, Mungyeong-si, Gyeongsangbuk-do, Korea	36.66186111	36°	39'	42.7"	N	128°	10'	25.9"	128.1738611	E	Y.G Choi	23.vi.2019	ON042202
Lep-812	*Leptoneta hogyegulensis*	Hogyegul cave: Byeoram-ri, Hogye-myeon, Mungyeong-si, Gyeongsangbuk-do, Korea	36.66186111	36°	39'	42.7"	N	128°	10'	25.9"	128.1738611	E	Y.G Choi	23.vi.2019	ON042203
Lep-814	*Leptoneta hogyegulensis*	Hogyegul cave: Byeoram-ri, Hogye-myeon, Mungyeong-si, Gyeongsangbuk-do, Korea	36.66186111	36°	39'	42.7"	N	128°	10'	25.9"	128.1738611	E	Y.G Choi	23.vi.2019	ON042204
Lep-815	*Leptoneta hogyegulensis*	Hogyegul cave: Byeoram-ri, Hogye-myeon, Mungyeong-si, Gyeongsangbuk-do, Korea	36.66186111	36°	39'	42.7"	N	128°	10'	25.9"	128.1738611	E	Y.G Choi	23.vi.2019	ON042205
Lep-816	*Leptoneta hogyegulensis*	Hogyegul cave: Byeoram-ri, Hogye-myeon, Mungyeong-si, Gyeongsangbuk-do, Korea	36.66186111	36°	39'	42.7"	N	128°	10'	25.9"	128.1738611	E	Y.G Choi	23.vi.2019	ON042206
Lep-817	*Leptoneta hogyegulensis*	Hogyegul cave: Byeoram-ri, Hogye-myeon, Mungyeong-si, Gyeongsangbuk-do, Korea	36.66186111	36°	39'	42.7"	N	128°	10'	25.9"	128.1738611	E	Y.G Choi	23.vi.2019	ON042207
Lep-818	*Leptoneta hogyegulensis*	Hogyegul cave: Byeoram-ri, Hogye-myeon, Mungyeong-si, Gyeongsangbuk-do, Korea	36.66186111	36°	39'	42.7"	N	128°	10'	25.9"	128.1738611	E	Y.G Choi	23.vi.2019	ON042208
Lep-819	*Falcileptoneta baegunsanensis*	Sanyang-ri, Hwacheon-eup, Hwacheon-gun, Gangwon-do, Korea	38.25138889	38°	15'	05.0"	N	127°	41'	02.1"	127.6839167	E	Y.G Choi	24.vii.2018	ON042209
Tel-01	*Telema* sp	Bettigul cave: San 117, Sayang-ri, Ssangchaek-myeon, Hapcheon-gun, Gyeongsangnam-do, Korea	35.61197222	35°	36'	43.1"	N	128°	15'	51.6"	128.2643333	E	Y.G Choi	12.xii.2020	ON042210
Tel-03	*Telema* sp	Bettigul cave: San 117, Sayang-ri, Ssangchaek-myeon, Hapcheon-gun, Gyeongsangnam-do, Korea	35.61197222	35°	36'	43.1"	N	128°	15'	51.6"	128.2643333	E	Y.G Choi	12.xii.2020	ON042211
Genbank	*Falcileptoneta baegunsanensis*														MT988000
Genbank	*Falcileptoneta boeunensis*														MT987997
Genbank	*Falcileptoneta chiakensis*														MT987982
Genbank	*Falcileptoneta coreana*														JN817068.1
Genbank	*Falcileptoneta hansanensis*														MT987985
Genbank	*Falcileptoneta juwangensis*														MT987993
Genbank	*Falcileptoneta odaesanensis*														MT987983
Genbank	*Falcileptoneta unmunensis*														MT987986
Genbank	*Longileptoneta weolakensis*														MT987984
Genbank	*Masirana bonghwaensis*														MT987996
Genbank	*Masirana flabelli*														MT987988
Genbank	*Masirana ilweolensis*														MT987994
Genbank	*Masirana ilweolensis*														MT987995

[Table S2. Number of species and individuals of each genus identified from collection data]

**Family**	**Genus**	**No. Morphospecies**	**No. Individuals**
Telemidae	*Telema*	1	2
Leptonetidae	*Falcileptoneta*	43	238
	Leptoneta	11	91
	Longileptoneta	17	74
	Masirana	2	6
Total	5	75	411

[Table S3. Multiple MOTUs result from species delimitation methods: ABGD, ASAP, GMYC, PTP, and bPTP]. Blanks represent a single MOTU.

Genus	Morphospecies	Multiple MOTUs				
		ABGD	ASAP	GMYC	PTP	bPTP
Falcileptoneta	*Falcileptoneta chiakensis* (6 sites)	5	6	6	6	6
	Falcileptoneta geumsanensis (4 sites)			3	4	4
	Falcileptoneta hansanensis (2 sites)		2		2	2
	Falcileptoneta juwangensis (2 sites)			2		
	Falcileptoneta maewhaensis (2 sites)	2	2	2	2	2
	Falcileptoneta naejangensis (2 sites)	3	3	3	3	3
	Falcileptoneta odaesanensis (7 sites)	2	2	2	2	2
	Falcileptoneta secula (2 sites)	2	2	2	2	2
	Falcileptoneta unmunensis (2 sites)			2	2	3
	Falcileptoneta sp2 (3 sites)	2	2	3	3	2
	Falcileptoneta sp3 (2 sites)			2	2	2
	Falcileptoneta sp4 (2 sites)			2	2	2
	Falcileptoneta sp16 (1 site)					2
	Falcileptoneta sp17 (3 sites)	2	2	2	2	2

Longileptoneta	*Longileptoneta byeonsanbando* (2 sites)			4	4	4
	Longileptoneta songniensis (2 sites)				2	2
	Longileptoneta gayaensis (2 sites)		2	2	2	2
	Longileptoneta sp3 (1 site)			2	3	3
	Longileptoneta sp4 (1 site)			2	2	2
	Longileptoneta sp6 (1 site)			2	2	2
Leptoneta	*Leptoneta chilbosanensis* (4 sites)	2	2	2	2	2
	Leptoneta namhensis (2 sites)		2	2	2	2
	Leptoneta paikmyeonggulensis (3 sites)			2	3	4
	Leptoneta taeguensis (7 sites)	6	6	7	7	7
	Leptoneta seogwipoensis (2 sites)				2	
	Leptoneta sp1 (1 site)				2	
Masirana	*Masirana ilweolensis* (6 sites)		2	2	2	5

[Table S4. Settings and results of Automatic Barcode Gap Discovery (ABGD) analysis] Jukes-Cantor substitution model (JC69), Kimura 2-parameter model (K2P), and Simple distance (p-distance) model.

**Settings**		**Prior intraspecific divergence (*P*)**								
**Model**	**Relative gap width**	**Partition**	0.0359	0.0215	0.0129	0.0077	0.0046	0.0028	0.0017	0.0010
JC69	X=1.5	Initial	72	84	72	72	72	72	72	72
		Recursive	82	84	88	95	97	112	121	150
K2P	X=1.5	Initial	92	92	92	92	92	92	92	92
		Recursive	-	94	97	102	104	119	127	155
Simple	X=1.5	Initial	91	91	91	91	91	91	91	91
		Recursive	-	95	95	96	102	108	116	116

[Table S5. Results of Assemble Species by Automatic Partitioning (ASAP) analysis]

**Model**											
**JC69**	Threshold dist.	0.026547	0.033246	0.0.039896	0.068631	0.063774	0.020355	0.028274	0.024250	0.018540	0.055199
	ASAP score	7.00	8.50	10.50	13.00	14.50	15.00	15.00	16.50	17.50	17.50
	No. Species	98	95	94	80	80	104	98	101	106	84
**K2P**	Threshold dist.	0.026560	0.033244	0.040004	0.020440	0.069028	0.028293	0.046979	0.063820	0.018549	0.025414
	ASAP score	9.50	10.50	14.00	16.00	17.00	17.50	18.00	18.50	19.50	19.50
	Species	98	95	94	104	80	98	88	80	106	100
**Simple**	Threshold dist.	0.026082	0.032514	0.038846	0.020081	0.065577	0.027747	0.018313	0.061137	0.023862	0.024972
	ASAP score	6.50	10.00	10.50	13.50	14.00	15.00	15.50	16.00	17.00	17.00
	Species	98	95	94	104	80	98	106	80	101	100
